# Growth hormone receptor disrupts glucose homeostasis via promoting and stabilizing retinol binding protein 4

**DOI:** 10.7150/thno.61192

**Published:** 2021-07-13

**Authors:** Jinxin Liu, Chenzhipeng Nie, Lamei Xue, Ying Yan, Shengnan Liu, Juan Sun, Mingcong Fan, Haifeng Qian, Hao Ying, Li Wang, Yan Li

**Affiliations:** 1 State Key Laboratory of Food Science and Technology, School of Food Science and Technology, Jiangnan University, Wuxi 214122, China;; 2 Chinese Academy of Sciences Key Laboratory of Nutrition, Metabolism and Food Safety, Shanghai Institutes for Biological Sciences, Chinese Academy of Sciences, 200031 Shanghai, China.

**Keywords:** GHR, RBP4, systemic insulin resistance, glucose homeostasis, hepatokine

## Abstract

**Rationale:** The molecular mechanisms underlying the pathogenesis of systemic insulin resistance in type 2 diabetes remain elusive. Growth hormone receptor (GHR) deficiency has long been known to improved insulin sensitivity. However, whether hepatic GHR overexpression or activation is a cause of insulin resistance is still unknown. The aim of this study was to identify the new role of GHR in systemic insulin resistance and explore the underlying mechanism.

**Method:** Different samples obtained from obese humans, *ob/ob* mice, *db/db* mice, high-fat diet (HFD)-fed mice and primary mouse hepatocytes were used to evaluate the correlations between GHR and metabolic disorders. Recombinant adeno-associated viruses encoding GHR and STAT5 and GHR knockout mice were used to investigate the roles of hepatic GHR in glucose homeostasis. Tissue H&E, Oil Red O and PAS staining were performed for histomorphological analysis. Gel filtration chromatography was employed for the separation of serum RBP4-TTR complexes. Plasmids (related to GHR, STAT5 and HIF1α), siRNA oligos (siGHR and siSTAT5), luciferase activity and ChIP assays were used to explore the potential mechanism of hepatic GHR.

**Results:** Here, we found that hepatic GHR expression was elevated during metabolic disorder. Accordingly, hepatic GHR overexpression disrupted systemic glucose homeostasis by promoting gluconeogenesis and disturbing insulin responsiveness in the liver. Meanwhile, hepatic GHR overexpression promoted lipolysis in white adipose tissue and repressed glucose utilization in skeletal muscle by promoting the circulating level of RBP4, which contributed to impaired systemic insulin action. A mechanistic study revealed that hepatic GHR disrupted systemic insulin sensitivity by increasing RBP4 transcription by activating STAT5. Additionally, overexpression of hepatic GHR promoted TTR transcriptional levels by enhancing the expression of HIF1α, which not only increased the protein stability of RBP4 but also inhibited renal clearance of RBP4 in serum.

**Conclusions:** Hepatic GHR overexpression and activation accelerated systemic insulin resistance by increasing hepatic RBP4 production and maintaining circulating RBP4 homeostasis. Our current study provides novel insights into the pathogenesis of type 2 diabetes and its associated metabolic complications.

## Introduction

Type 2 diabetes mellitus (T2DM) has become a major global public health concern and is caused by severe systemic insulin resistance and impaired insulin sensitivity. Tissue crosstalk through autocrine, paracrine and endocrine mechanisms clearly not only plays critical roles in regulating energy homeostasis but also contributes to the development of insulin resistance 1-3. Recently, a growing body of evidence suggests a pivotal involvement of the liver as a metabolic modulator that dictates insulin sensitivity of multiple peripheral tissues or the central nervous system by secreting hepatokines in response to nutrient overabundance and deficiency 1, 4, 5. Manipulating the levels of hepatokine in circulation could improve systemic insulin resistance and glucose tolerance in diabetic mammals, indicating that hepatokine is required for maintaining normal glucose homeostasis in T2DM 6-9. Thus, a better understanding of the relationship and underlying mechanisms between insulin resistance and hepatokines is needed.

Retinol-binding protein 4 (RBP4) is a metabolic cytokine that has meaningful effects on glucose and lipid homeostasis. RBP4 was originally identified as a specific transport protein for retinol in circulation 10. However, RBP4 is generally considered a hepatokine that acts in an endocrine manner because the liver is the principal source of circulating RBP4 levels, while skeletal muscle and adipose tissue are the major targets of RBP4 11-13. Elevated RBP4 levels were observed in insulin-resistant mice and humans with obesity or T2DM 12, 13. In agreement with the regulatory role of RBP4 in systemic insulin resistance, mice overexpressing RBP4 displayed severe insulin resistance or glucose intolerance 14, 15, while mice with reduced RBP4 levels exhibited increased insulin sensitivity and improved glucose homeostasis 16. However, a previous study also noted that liver-secreted RBP4 did not impair glucose homeostasis in mice 17. What needs illustration is that mice with systemic insulin resistance display not only elevated circulating RBP4 levels but also attenuated renal clearance of RBP4, which is associated with the circulating level of transthyretin (TTR)-RBP4 formation that may reduce the glomerular filtration and renal catabolism of RBP4 10, 18, 19. Although the role of RBP4 in glucose metabolism has been extensively studied, the regulatory networks of RBP4 in obesity, insulin resistance and diabetes are still poorly understood.

Growth hormone receptor (GHR) is a member of the cytokine family of receptors that, upon binding to growth hormone (GH), activates tyrosine kinase Janus kinase 2 (JAK2), which in turn phosphorylates its downstream targets, including the signal transducer and activator of transcription (STAT) pathway 20-22. Recently, the role of GHR in metabolic regulation has gained great attention and is widely involved in energy expenditure, fuel utilization and metabolism homeostasis 23-26. GHR expression in adipose tissue has been reported to be decreased in subjects with obesity 27. However, changes in hepatic GHR expression in response to fasting or genetic and diet-induced mouse models of T2DM have not been examined. Additionally, GHR knockout mice are characterized by smaller adult body size, extended longevity and remarkedly elevated whole-body insulin sensitivity 28. However, it is currently unclear whether hepatic GHR overexpression or activation is a cause of insulin resistance in commonly used mouse models of T2DM. Furthermore, since the liver functions as an endocrine organ in systemic insulin sensitivity, whether hepatic GHR is can affect hepatokine-regulated interorgan communication, thereby being involved in the pathogenesis of insulin resistance is unknown.

## Materials & methods

### Animal experiments and human study

Male C57BL/6J mice aged 8 to 10 weeks were purchased from the Shanghai SLAC Laboratory Animal Company (Shanghai, China). Male *ob/ob* and *db/db* mice (C57BL/6 background) aged 8 to 10 weeks were obtained from the Model Animal Research Center of the Nanjing University (Nanjing, China). The HFD-induced obese mice were generated using C57BL/6 mice (aged 8 to 10 weeks) fed *ad libitum* with normal chow diet (NCD) or HFD (D12492, Research Diets) for 12 weeks. Heterozygous GHR knockout (GHR^+/-^) mice (C57BL/6 background) were purchased from Biocytogen Company (Beijing, China), the GHR knockout (GHR-KO) and littermate wild-type (GHR-WT) mice were derived from heterozygous (GHR^+/-^) mating pairs. The recombinant adeno-associated viruses (AAV) were used for the overexpression of GHR and knockdown of STAT5 expression. The liver-specific GHR overexpression mice and corresponding control mice were generated via the injection of recombinant adeno-associated virus serotype 8 (AAV8) gene transfer vectors containing a liver-specific promoter combination (albumin promoter) with either mouse GHR sequence (AAV-GHR) or empty as vector control (AAV-GFP) (Hanbio Biotechnology Co., LTD, Shanghai, China). AAV-GFP or AAV-GHR were administered by lateral tail vein injection at a dose of 1×10^12^ vg/mL in a total volume of 100 μl/mice at the beginning of the experiment. Recombinant AAV8 vectors encoding mouse GHR (NM_010284.3) under the control of albumin promoter (a liver-specific promoter) were generated by Hanbio Biotechnology Co. LTD. (Shanghai, China). Furthermore, the AAV-shRNA was employed to knock down hepatic STAT5 expression of mice. The AAV-mediated shRNA that targets STAT5 was cloned into AAV9 vectors downstream to a U6 promoter, and control AAV9 vectors containing either a GFP (AAV-shNC) or a scrambled shRNA expression cassette (AAV-shSTAT5). AAV-shNC or AAV-shSTAT5 were also administered by lateral tail vein injection at a dose of 1×10^12^ vg/mL in a total volume of 100 μl/mice in our experiment. The shRNA sequence specific for mouse STAT5 was 5'-GGGACCTGAATTACCTCATAT-3'. The RBG of mice was measured every 5 days and the mice samples were harvested 40 days after AAV injection. All the mice were housed in a specific pathogen-free animal facility illuminated with a 12 h light/12 h dark cycle at a constant temperature of 23 °C ± 2 °C with 55% ± 5% humidity. All the protocols of the study were reviewed and approved by the Animal Ethics Committee of Jiangnan University (JN. No 20180915c0180630 [188] and JN. No 20190930c0600630 [247]). All efforts were made to minimize animal discomfort. The mice were anesthetized with sodium pentobarbital before sacrifice as described in our previous study 29. The FBG or RBG were detected from the tail vein using a portable blood glucose meter (FreeStyle Optium Neo, Abbott) before harvest. Tissues were harvested without restricting the mice to food or water, unless indicated, snap-frozen in liquid nitrogen immediately after resection and stored at -80 °C. Especially, the serum was collected and stored at -80 °C after centrifugation, and partial liver and Ing were fixed at 4% paraformaldehyde/phosphate buffered saline (PBS) (pH = 7.2) or absolute ethyl alcohol for 24-36 h for further histological analysis. For insulin signaling analysis, mice were fasted for 6 h and intraperitoneally (i.p.) injected with 0.75 U/kg of human insulin (Novolin^®^ 30R Penfill^®^, Novo Nordisk) or saline, 10 min after injection, the liver, Ing and GAS of mice were harvested and snap-frozen in liquid nitrogen for further immunoblot analysis.

In order to analyze the expression levels of GHR and RBP4 in obese humans, liver samples of control subjects were obtained via biopsy during laparoscopic cholecystectomy. Liver samples of obese patients were obtained via biopsy during bariatric surgery. The characteristics of human subjects are shown in Figure S1A and Table S1. All the human samples were gifted by Dr. Jingjing Jiang and the human study was reviewed and approved by the Human Research Ethics Committee of Zhongshan Hospital. Written informed consent was obtained from each subject prior to study participation.

### Tolerance tests

Glucose tolerance tests and pyruvate tolerance tests were carried out on mice by i.p. injection with 1 or 2 g/kg body weight of D-glucose or pyruvate (Sigma-Aldrich) after a 16 h fast. The insulin tolerance tests were also performed on 6 h fasted mice with the i.p. injection of regular human insulin (Novolin^®^ 30R Penfill^®^, Novo Nordisk) at a dose of 0.75 U/kg body weight. Blood glucose levels were detected from the tail vein before injection and at 15, 30, 60 and 120 min after injection using a portable blood glucose meter (FreeStyle Optium Neo, Abbott).

### ELISA analysis

Total triglyceride (TG) concentrations of the serum and liver were measured by a micro-plate reader (Bio-Tek, USA) using corresponding enzymatic assays (#90-63701, Wako). Especially, the concentrations of hepatic TG, extracted with chloroform/methanol (2:1, v/v) as reported in our previous study 29, were also determined using the above commercial enzymatic assay. The total lipid content in the liver was determined gravimetrically after extraction. Moreover, the serum RBP4 concentrations were respectively carried out on humans (#DRB400) and mice (#DRBP40) using corresponding RBP4 Quantikine ELISA Kit (R&D Systems, USA). Furthermore, the serum levels of insulin were measured by ELISA kit (Millipore, USA). Total glycogen in the liver was measured according to the methods of Glycogen Assay Kit (BioVision, USA). In addition, the concentrations of serum GH and IGF-1 were also carried out on humans using corresponding enzymatic assays, respectively (Huijia Biotechnology, China).

### Histological analysis

Tissue H&E, Oil Red O or PAS staining of liver and Ing were respectively performed after tissue fixation for 24-36 h as described in a previous study 30. Briefly, the fixed specimens (liver and Ing) were embedded in paraffin and were sectioned at 5 μm for H&E staining. For Oil Red O staining, the fixed livers were sequentially equilibrated in 10% and 20% sucrose for 12 h at 4 °C, respectively, and then the partial livers were embedded in optimal cutting temperature compound and frozen at -20 °C for next 20 μm thick cryostat sections and Oil Red O staining according to the manufacturer's protocol. Different from the above fixed method for PAS staining, the partial livers were harvested and fixed in absolute ethyl alcohol for 36 h before paraffin embedding, and then 5 μm liver sections were obtained and stained for histological analysis. Finally, all the stained slides were sealed with resinene after dehydrated to transparency and visualized by inverted light microscopy (ZEISS Axio Vert A1, Germany).

### Gel filtration chromatography

Gel filtration chromatography was employed for the separation of serum RBP4-TTR complexes as described in previous study 19. Briefly, the serum (0.5 ml) was loaded onto a HiLoad 16/600 Superdex 75 pg column connected to an ÄKTA purifier (GE Healthcare). Protein complexes were separated by passing phosphate-buffered saline (0.01M PBS) at 1.0 ml/min for 150 min at room temperature. Eluting proteins were detected by absorbance at 280 nm and collected according to the ultraviolet absorption peak of fractions. Finally, the condensed proteins were redissolved with 50 μl RIPA lysis buffer (Beyotime, China) for further immunoblot analysis.

### Primary mouse hepatocyte isolation and cell culture

Male C57BL/6J mice aged 8-10 weeks were anaesthetized with sodium pentobarbital (100 mg/kg, Sigma-Aldrich) and the portal vein was cannulated under aseptic conditions. The liver was firstly perfused with 30 ml 1x Hanks' Balanced Salt Solution (1x HBSS) (Sigma-Aldrich) containing 2.28 mg EGTA at 37 ℃, then digested with 50 ml 1x HBSS containing 0.05% collagenase type I (Sigma-Aldrich) and 27.75 mg CaCl_2_ at 37 ℃. The digested mouse liver was then aseptically removed to a sterile 10-cm cell culture dish and dispersed with DMEM (Gibco) containing 10% fetal bovine serum (FBS, Gibco). The isolated mouse hepatocytes were filtrated through a cell strainer (100 μm) into a 50-ml centrifuge tube and centrifuged at 500 g for 5 min at 4 ℃. The hepatocytes were then resuspended with 12.5 ml DMEM containing 10% FBS. And cold 12.5 ml Percoll solution, containing 11.25 ml Percoll (GE Healthcare) and 1.25 ml 10x HBSS, were added to isolate the hepatocytes. The homogeneous cell suspension was centrifuged at 1000 g for 10 min at 4 ℃, the supernatant cells were removed and the centrifugated cells were washed with 0.01M PBS and counted after suspension. The isolated mouse hepatocytes were seeded at a density of 5×10^5^ cells per well in rat tail collagen type I-coated 6-well plates for 5 h at 37 ℃ and cultured in DMEM containing 10% FBS and 1% penicillin/streptomycin (PS, streptomycin 100 μg/ml, penicillin 100 U/ml, Gibco) for further experiments. In addition, the human HepG2 cells were maintained at 37 °C in 5% CO_2_ in DMEM containing 10% FBS and 1% PS for further experiments as indicated.

For glucose treatment, the primary mouse hepatocytes were harvested after culturing for 24 h in DMEM with high glucose (4.5 mg/ml, Con), no glucose (NG) or low glucose (1.0 mg/ml, LG), respectively. Regarding PA (Sigma-Aldrich) treatment, PA was dissolved in isopropanol at a concentration of 40 mM as stock solution. The isopropanol treatment was used as control group. The final treatment concentrations were all 200 mM. Before PA treatment, primary hepatocytes were fasted overnight in DMEM medium containing 0.5% BSA and then treated for 4 h before harvest.

### Plasmids, RNA oligonucleotide and luciferase reporter assays

The full-length GHR were obtained by PCR amplification of the cDNA from mouse liver and cloned into pBabe-Puro vector with BamHI/EcoRI restriction sites to generate GHR expression plasmid. The STAT5 expression plasmid was purchased from Addgene. The pcDNA3.1-HIF1α-DM was used as described in our previous study 31. The control siRNA, siGHR and siSTAT5 RNA oligos were purchased from Genepharma (Shanghai, China). The luciferase assays were performed as previously reported with modification 32. Briefly, for the construction of luciferase reporter plasmids, the pGL-3 basic plasmid was used as the control. Enhancer or possible promoter sequences of STAT5 and HIF1α containing SRE site or HRE site were amplified by using human genomic DNA and inserted into pGL-3 vector or pTK109-luc vector. The plasmids were cotransfected into HepG2 cells using Lipofectamine™ 2000 Transfection Reagent (Invitrogen) and Opti-MEM (Invitrogen) as per the instructions. Luciferase activity was performed using the Dual Luciferase Reporter Assay System (Promega) after cell transfection for 24 h or 48 h. For hypoxic conditions, cells were incubated in a hypoxic chamber with 1% O_2_, 5% CO_2_ and 94% N_2_ for 24 h after 24 h transfection. The PCR primer sequences and siRNA information are shown in Table S2.

### SDS-PAGE and Western blotting analysis

Western blot analysis was performed as previously described 39. Briefly, the tissues or cells were lysed in RIPA lysis buffer (Beyotime, China) supplemented with phosphatase and protease inhibitors (Roche, Switzerland), followed by 10 min boiling and centrifugation to obtain the supernatant for next experiment. Samples containing equal amounts of protein were separated on 10% SDS-PAGE and subsequently transferred to PVDF membranes. The membranes were incubated with various antibodies at 4 ℃ overnight. The primary antibodies for GHR (#sc-137185), PEPCK (#sc-32879), G6Pase (#sc-25840) (Santa Cruz Biotechnology, 1:1000), p-IR (#3024), IR (#3025), p-Akt (Ser473) (#9271), Akt(#9272), p-Foxo1 (#9464), Foxo1(#2880), p-GSK3β (#5558), GSK3β (#9315), HSL (#4107), ATGL(#2138), STAT5 (#94205), p-STAT5 (#9359) (Cell Signaling Technology, 1:1000), GAPDH (#10494-1-AP, 1:5000), PDH (#18068-1-AP), RBP4 (#11774-1-AP), TTR (#11891-1-AP), β-actin (#20536-1-AP) (ProteinTech Group, 1:1000), PDK4 (#ab38242), HIF1α (#ab228649) (Abcam, 1:1000), p-PDH (#ABS194, Merck Millipore, 1:1000) were used, and secondary antibodies including Peroxidase-AffiniPure Goat Anti-Rabbit IgG (#111-035-003) and Peroxidase-AffiniPure Goat Anti-Mouse IgG (#115-035-003) (Jackson ImmunoResearch, 1:2000-1:5000) were also used for Western blot analysis. Finally, the blots were visualized by the C-Digit^®^ digital chemiluminescence imager (LI-COR, USA). Especially, the pretreatment of serum for SDS-PAGE was described as previous study 33. Briefly, the low molecular weight and low abundance serum proteins were enriched with 60% acetonitrile, and the supernatant was concentrated by CentriVap^®^ Centrifugal Vacuum Concentrator (Labconco, USA), then the precipitate was redissolved with 100 μl ddH_2_O and processed with Protein Deglycosylation Mix II (NEB, USA). Finally, the deglycosylated proteins were redissolved with above mixed RIPA lysis buffer and denatured in the same manner. The serum proteins were separated on 10% SDS-PAGE for Coomassie Brilliant Blue (CBB) staining, and Western blotting analysis were also performed.

### Quantitative real-time PCR (qRT-PCR) analysis

The qRT-PCR analysis was performed as described in our previous reports 29, 34. Briefly, total RNAs were extracted from tissues and cells using TRIzol reagents (Invitrogen, USA) according to the manufacturer's protocol and quantified by Nanodrop (Thermo Fisher Scientific, USA). Synthesis of cDNAs was executed using the Prime Script RT system (Takara, Japan). The qRT-PCR analysis was performed using FastStart Universal SYBR Green Master (ROX) (Roche, Switzerland) on an ABI 7900 RT-PCR system (Applied Biosystem, USA). 18S or GAPDH was used as the internal control to normalize the data to determine the relative expression of the target genes by using the 2^-ΔΔCt^ method. The primer sequences used for qRT-PCR analysis are listed in Table S3. In addition, the heatmap for qRT-PCR analysis and the partial least squares-discriminant analysis (PLS-DA) of them were carried out by MetaboAnalyst software (version 4.0; https://www.metaboanalyst.ca/).

### Chromatin immunoprecipitation (ChIP) assay

ChIP assays were executed in AAV-GHR mice using the EZ Magna ChIP kit (Millipore, USA) according to the manufacturer's protocol. Anti-STAT5 or anti-HIF1α was used to precipitate DNA fragments, respectively. PCR was carried out to analyze the binding site of STAT5 or HIF1α. CCND1 SRE or VEGF HRE was respectively used as a positive control. A primer set for non-SRE or non-HRE region was used as a negative control. ChIP primers are presented in Table S4.

### Statistical analysis

All experiments were performed at least in triplicate, and representative data were shown. The data were shown as mean ± standard deviation (SD) for each group. Student's *t*-test was performed to analyze the difference between two groups, and one-way ANOVA was used to analyze the difference between three groups. The significance was presented as *, *p* < 0.05; **, *p* < 0.01; ***, *p* < 0.001; and no significant difference was presented as ns. All statistical analyses were performed using GraphPad Prism 8.0 (GraphPad Software, La Jolla, USA).

## Results

### GHR is elevated in the liver during metabolic disorder

To evaluate the role of hepatic GHR in metabolic regulation, we first determined the mRNA expression of GHR in the liver of obese subjects, which were collected from a cohort of age-matched healthy male subjects (BMI: 20.96 ± 3.41 kg/m^2^) and patients with obesity (BMI: 41.84 ± 4.89 kg/m^2^) (Table S1). Moreover, the serum TG level was significantly enhanced, while the serum GH level was reduced in obese patients compared to control subjects. Interestingly, there was no significant change in serum IGF-1 levels (Table S1). Interestingly, the serum GH level was reduced while hepatic GHR expression was increased in obese patients compared to control subjects. One possible explanation was that, as previously reported, the GH level was negatively correlated with body mass index (BMI) and the secretion of endogenous GH in obese people was lower than the secretion of endogenous GH in normal people, while hepatic GHR expression was upregulated in obese people to maintain serum IGF-1 levels within the normal range, which might be a protective mechanism 35, 36.

**Figure 1:**
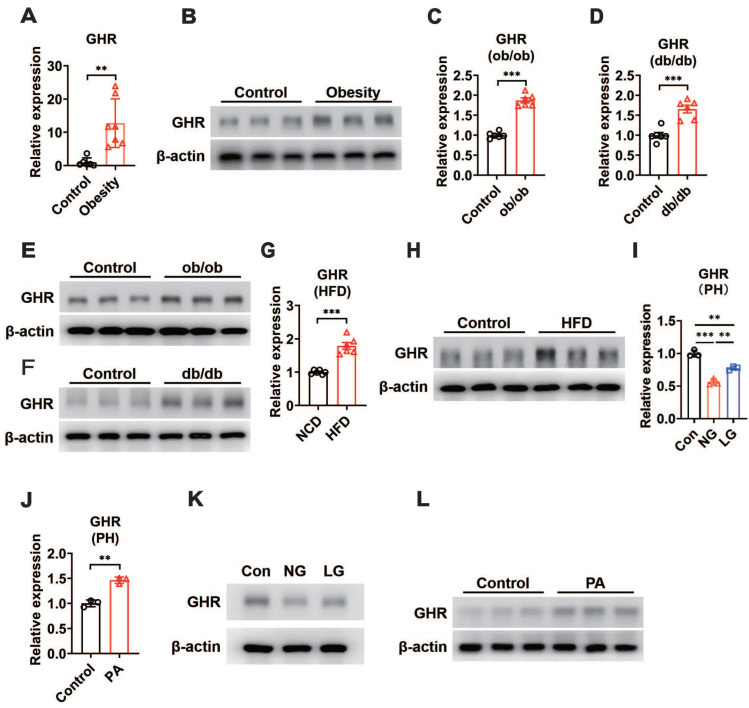
** GHR is up-regulated in the liver during metabolic disorder.** (A and B) Relative mRNA levels (A, n=6-7) and protein levels (B) of GHR determined by qRT-PCR or Western blots in the livers of humans. (C and D) Relative mRNA levels of GHR were determined by qRT-PCR in the livers of ob/ob mice (C, n=6) or db/db mice (D, n=6), respectively. (E and F) Protein levels of GHR were determined by Western blots in the livers of ob/ob mice (E) or db/db mice (F), respectively. (G and H) Relative mRNA levels (G, n=6) and protein levels (H) of GHR determined by qRT-PCR or Western blots in the livers of NCD-fed or HFD-fed mice, respectively. (I and J) Relative mRNA levels of GHR determined by qRT-PCR in primary hepatocytes (PH) cultured in high glucose DMEM (Con), no glucose DMEM (NG) and low glucose DMEM (LG) (I) or treated with palmitic acid (PA) (J), respectively. (K and L) Protein levels of GHR determined by Western blots in primary hepatocytes (PH) cultured in high glucose DMEM (Con), no glucose (NG) DMEM and low glucose (LG) DMEM (K) or treated with palmitic acid (PA) (L), respectively. Data are expressed as the mean ± SD. **p < 0.01; ***p < 0.001 (Student's *t*-test or one-way ANOVA).

In addition, our results revealed an increase in hepatic GHR mRNA and protein levels in obese patients compared with control subjects (Figure 1A-B). We next determined whether hepatic GHR levels were altered in metabolic disorder mouse models. Consistently, we found that hepatic GHR expression was markedly elevated in leptin-deficient *ob/ob* mice and leptin receptor-deficient *db/db* mice at both the mRNA and protein levels (Figure 1C-F). The levels of hepatic GHR were also elevated in high-fat diet-induced obesity (Figure 1G-H). The levels of fasting blood glucose (FBG) were significantly increased in obese individuals (Figure S1A), as well as all the metabolic disorder mouse models (Figure S1B-D). Based on these findings, we preliminarily speculated that the expression of hepatic GHR might be associated with abnormal metabolic homeostasis. To further explore the potential mechanism, primary hepatocytes were isolated from wild-type mice and cultured in DMEM with glucose or fatty acid (PA) treatment to investigate whether GHR expression was affected by high concentrations of glucose or fatty acids. Intriguingly, the results showed a positive correlation between GHR expression and glucose concentration in primary hepatocytes (Figure 1I), and elevated GHR mRNA expression was also observed in PA-treated primary hepatocytes (Figure 1J), indicating that both high concentrations of glucose and fatty acids could modify the transcription level of GHR. Consistently, the corresponding changes in GHR translation levels were also presented (Figure 1K-L). Taken together, multiple lines of evidence indicate that elevated GHR is associated with abnormal metabolic homeostasis.

### Hepatic GHR overexpression disrupts glucose homeostasis

**Figure 2:**
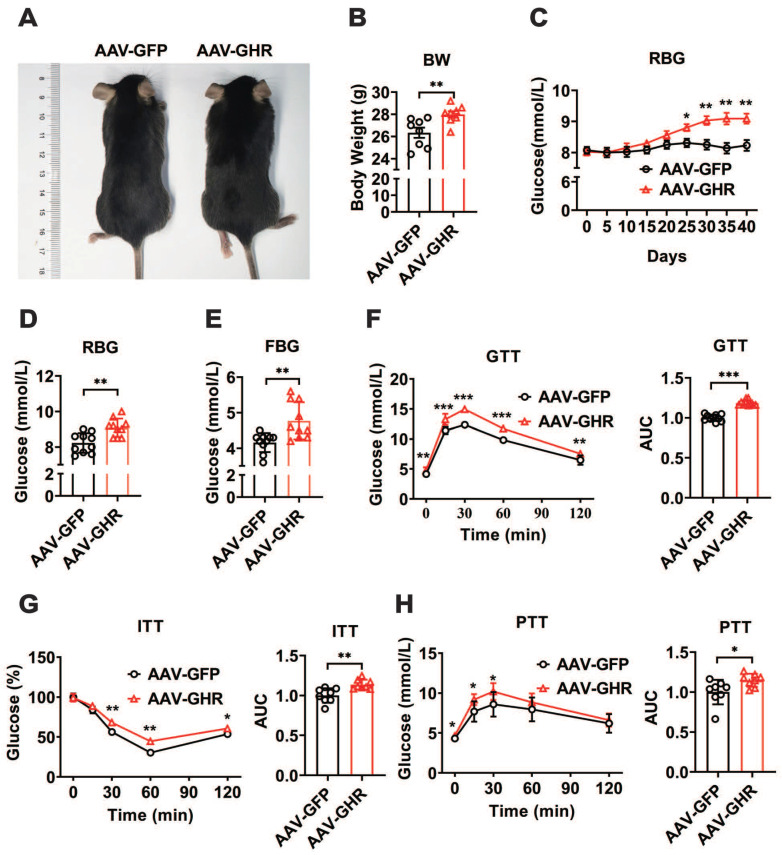
** Hepatic GHR overexpression disrupts glucose homeostasis.** (A and B) Representative photograph (A) and body weight (B, n=8) of mice 40 days after AAV-GFP or AAV-GHR injection. (C) Time course of random blood glucose (RBG) levels of mice after AAV-GFP or AAV-GHR injection (n=10). (D and E) The RBG (D, n=10) and FBG (E, n=10) levels of AAV-GFP or AAV-GHR infected mice before sample harvest. (F-H) Glucose tolerance tests (GTT, F, n=10), insulin tolerance tests (ITT, G, n=8) and pyruvate tolerance tests (PTT, H, n=8) performed in mice infected with AAV-GFP or AAV-GHR, and the area under the curve (AUC) data for GTT (F), ITT (G) and PTT (H) tests were calculated, respectively. Data are expressed as the mean ± SD. *p < 0.05; **p < 0.01; ***p < 0.001 (Student's* t*-test).

To further confirm the relationship between GHR and metabolic homeostasis, a mouse model of liver-specific GHR overexpression was established by adeno-associated virus tail injection. Interestingly, compared to the body weight of GHR-GFP mice, the body weight of AAV-GHR mice was significantly increased 40 days after AAV-GHR injection (Figure 2A-B). Moreover, the random blood glucose (RBG) level gradually rose in AAV-GHR mice, and a significant difference was observed 25 days after AAV injection (Figure 2C). As expected, both RBG and FBG levels were dramatically increased 40 days after AAV injection (Figure 2D-E), suggesting that overexpression of hepatic GHR alters glucose metabolism. In contrast, mice with whole-body KO of GHR (GHR-KO mice), which were shorter and leaner than the control mice (Figure S2A-B), exhibited decreased RBG and FBG levels (Figure S2C-D). Furthermore, strong evidence from GTT, ITT and PTT was analyzed to determine the validity of our hypothesis. As expected, GTT and ITT presented an impaired glucose disposal rate and reduced insulin sensitivity in AAV-GHR mice (Figure 2F-G). The observation above prompted us to evaluate the effect of GHR on hepatic gluconeogenesis. Hepatic GHR overexpression significantly increased gluconeogenesis and hepatic glucose output in mice, as indicated by PTT (Figure 2H). Based on these data, we proposed that hepatic GHR overexpression disrupted glucose homeostasis.

### Hepatic GHR overexpression promotes insulin resistance and gluconeogenesis in the liver

**Figure 3:**
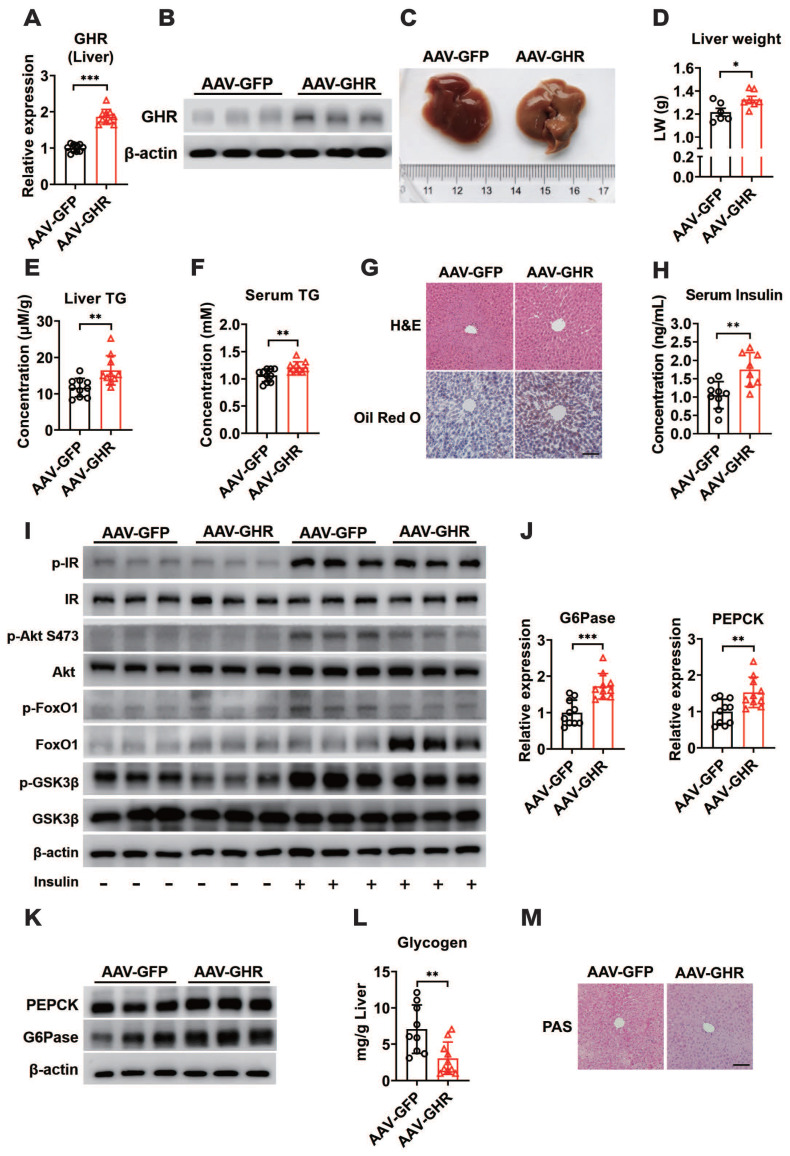
** Hepatic GHR overexpression results in insulin resistance and gluconeogenesis in the liver.** (A and B) Relative mRNA levels (A, n=10) and protein levels (B) of GHR in the livers of AAV-infected mice. (C and D) Representative liver photograph (C) and liver weight (D, n=6-7) of AAV-infected mice. (E and F) The TG levels in the livers (E, n=10) and serum (F, n=10) of AAV-infected mice. (G) Representative images of H&E staining (up) and Oil Red O staining (down) of liver sections from AAV-GFP (left) or AAV-GHR (right) mice. Scale bar, 500 μm. (H) The serum insulin levels of AAV-infected mice (n=8-9). (I) Western blots analysis of phosphorylated key molecules of the insulin signaling pathway in the liver of AAV-infected mice after insulin administration. (J and K) Relative mRNA levels (J, n=10) and protein levels (K) of gluconeogenesis-related genes or proteins in the livers of AAV-infected mice, respectively. (L) The amount of glycogen of AAV-infected mice normalized based on liver weight (n=9-10). (M) Representative images of PAS staining of liver sections from AAV-GFP (left) or AAV-GHR (right) mice. Scale bar, 500 μm. Data are expressed as the mean ± SD. *p < 0.05; **p < 0.01; ***p < 0.001 (Student's* t*-test).

Based on abnormal glucose metabolism, we further confirmed the expression of hepatic GHR, which was observably overexpressed in the livers of AAV-GHR mice (Figure 3A-B). The pictorial diagram of the liver showed abnormal morphology, and the liver weight was increased in AAV-GHR mice (Figure 3C-D). The triglyceride (TG) levels were determined to estimate the effects of GHR on liver metabolism. Elevated TG levels were observed in both the liver and serum of AAV-GHR mice (Figure 3E-F). Hepatic histomorphological analysis was also executed to confirm lipid accumulation by hematoxylin and eosin (H&E) and Oil Red O staining. As shown in Figure 3G, H&E and Oil Red O staining displayed increasing vacuoles and lipid droplets in the liver vision of AAV-GHR mice. Interestingly, opposite results were observed in the supplementary loss-of-function experiments. Since GHR was knocked out (Figure S3A-B), the TG levels were improved in the liver and serum of GHR-KO mice (Figure S3C-D). Moreover, decreased vacuoles and lipid droplets were dispersed in the liver of GHR-KO mice through histomorphological analysis (Figure S3E). These intuitive results suggested that hepatic GHR was also involved in lipid metabolism, which increased the serum lipid levels and accentuated hepatic lipid accumulation.

Since insulin is a critical hormone for maintaining glucose homeostasis, serum insulin levels were evaluated and found to be increased in AAV-GHR mice (Figure 3H), while serum insulin levels declined significantly in GHR-KO mice (Figure S3F), suggesting that hepatic GHR overexpression might repress insulin sensitivity and impair insulin signaling. To further understand the molecular change underlying the effect of hepatic GHR overexpression on glucose homeostasis and lipid metabolism, the expression of proteins involved in the insulin signaling pathway was investigated. As shown in Figure 3I, the insulin-induced phosphorylation of IR, Akt (S473), FoxO1 and GSK3β was reduced in the liver of AAV-GHR mice compared with AAV-GFP mice, presenting an impaired insulin signaling, while insulin signaling was improved in the liver of GHR-KO mice (Figure S3G). In addition, increased blood glucose levels trigger the secretion of insulin, which represses gluconeogenesis, provokes glycogen synthesis and induces lipogenesis in the liver 37. Accordingly, the expression of key gluconeogenic genes, G6Pase and PEPCK, was enhanced at both the mRNA and protein levels in the livers of AAV-GHR mice (Figure 3J-K), while these genes were suppressed in the livers of GHR-KO mice (Figure S3H-I). Furthermore, both glycogen content analysis with the glycogen assay kit and periodic acid Schiff (PAS) staining results showed that the glycogen level was decreased in the livers of AAV-GHR mice (Figure 3L-M). However, similar results were also observed in the livers of GHR-KO mice (Figure S3J-K). Thus, we proposed that hepatic GHR overexpression induced insulin resistance and gluconeogenesis in the liver.

### Hepatic GHR overexpression improves metabolic homeostasis of skeletal muscle and white adipose tissue

**Figure 4:**
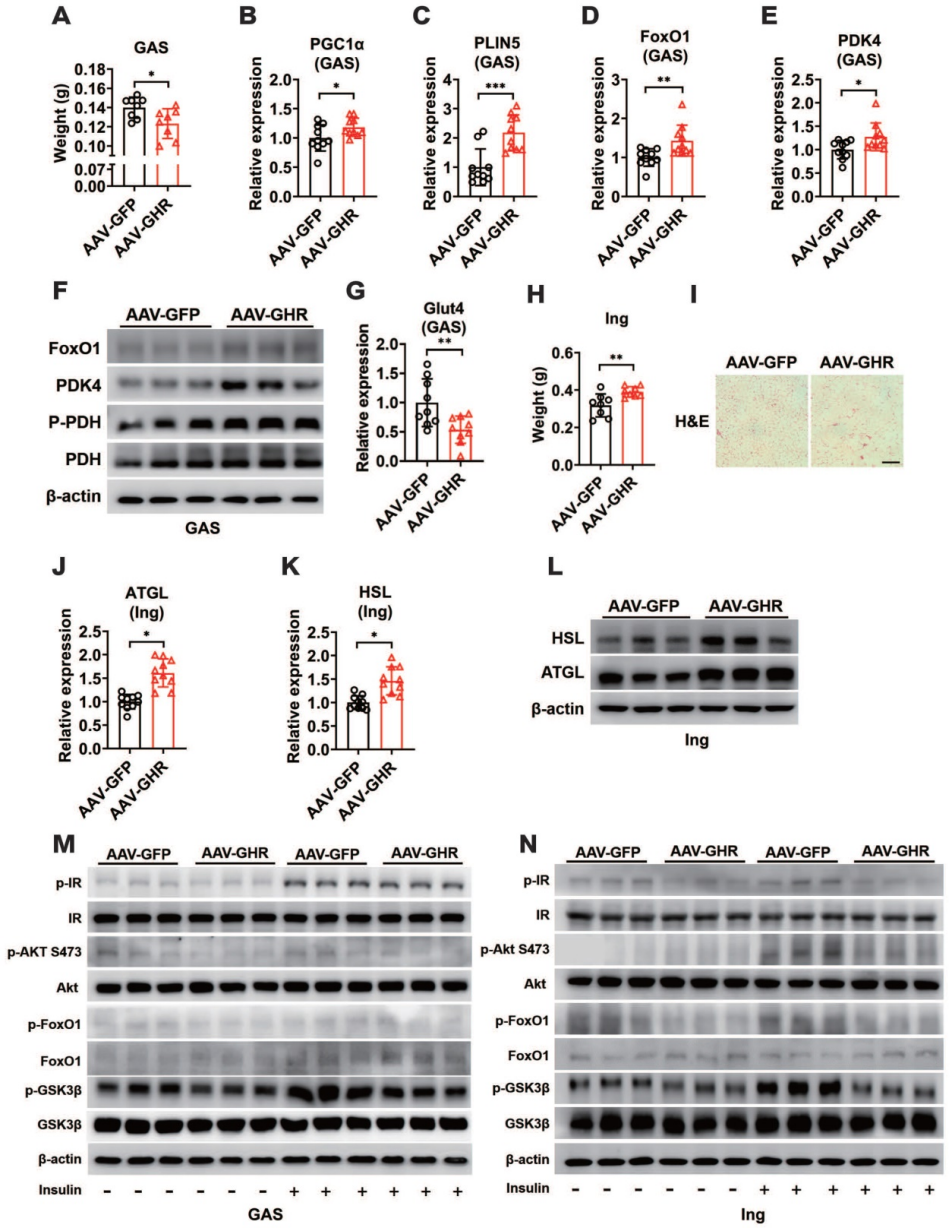
** Hepatic GHR overexpression improves metabolic homeostasis of skeletal muscle and white adipose tissue.** (A) The GAS weight of AAV-infected mice (n=8). (B-E) Relative mRNA levels of PGC1α (B), PLIN5 (C), FoxO1 (D) and PDK4 (E) in the GAS of AAV-infected mice (n=10). (F) Western blots analysis of FoxO1, PDK4, p-PDH and PDH in the GAS of AAV-infected mice. (G) Relative mRNA levels of Glut4 in the GAS of AAV-infected mice (n=9). (H) The Ing weight of AAV-infected mice (n=8). (I) Representative images of H&E staining of Ing sections from AAV-GFP (left) or AAV-GHR (right) mice. Scale bar, 500 μm. (J and K) Relative mRNA levels of ATGL (J, n=10) and HSL (K, n=10) in the Ing of AAV-infected mice. (L) Western blots analysis of HSL and ATGL in the Ing of AAV-infected mice. (M and N) Western blots analysis of phosphorylated key molecules of the insulin signaling pathway in the GAS (M) or Ing (N) of AAV-infected mice after insulin administration, respectively. Data are expressed as the mean ± SD. *p < 0.05; **p < 0.01; ***p < 0.001 (Student's* t*-test).

As important sites for fuel utilization and energy storage, we hypothesized that hepatic GHR also contributed to maintaining normal metabolic balance in skeletal muscle and white adipose tissue (WAT) through tissue communication. We first examined the expressions of GHR in skeletal muscle. Interestingly, no difference in the expression of GHR was observed in the gastrocnemius (GAS) muscle of AAV-GHR mice compared with AAV-GFP mice (Figure S4A-B). However, shrunken GAS muscle was present in AAV-GHR mice (Figure 4A, S4C), suggesting that a harmful effect might be induced by hepatic GHR overexpression. Hence, we next examined whether intramuscular fuel utilization was changed. Increased mRNA expression of PGC1α and PLIN5, two key genes involved in oxidative metabolism, was observed in the GAS muscles of AAV-GHR mice (Figure 4B-C), suggesting that more fatty acids or glucose might be used in the GAS muscles. Since the FoxO1-PDK4-PDH axis plays a crucial role in fuel usage in skeletal muscle 2, the mRNA and protein levels of either FoxO1 or PDK4 were increased in the GAS muscle of AAV-GHR mice (Figure 4D-F). Accordingly, the phosphorylation of PDH was also elevated in the GAS muscle of AAV-GHR mice (Figure 4F), indicating that glucose utilization is suppressed in the GAS muscle. In addition, the mRNA expression of Glut4 was reduced in the GAS muscle of AAV-GHR mice (Figure 4G), suggesting that glucose uptake was suppressed because of hepatic GHR overexpression.

As more fatty acids were taken advantage of by skeletal muscle, we speculated that the storage or lipolysis of fat might be changed in white adipose tissue. We first examined the expression of GHR in inguinal white adipose tissue (Ing). Accordingly, there was no significant difference in the expression of GHR in the Ing of AAV-GHR mice (Figure S4D-E). However, more lipid accumulation was observed in the Ing of AAV-GHR mice than in the Ing of AAV-GFP mice (Figure 4H, S4F), and abnormal lipid accumulation was also confirmed by H&E staining, which revealed enlarged adipocytes in the field of Ing vision of AAV-GHR mice (Figure 4I). Fat accumulation might be a protective mechanism to reduce glucose overloading in hyperglycemic environments. However, the expression of ATGL and HSL was also elevated at both the mRNA and protein levels in the Ing of AAV-GHR mice in comparison with AAV-GFP mice (Figure 4J-L), suggesting that lipolysis was enhanced, which might serve as a protective mechanism to reduce lipid overload. Moreover, more free fatty acids could be released into the circulation, which in return would provide sufficient substrates for hepatic gluconeogenesis and glucose output, as well as fatty acid oxidation in skeletal muscle. Given the abnormal metabolic homeostasis in both skeletal muscle and white adipose tissue, we speculated that insulin sensitivity might be changed by hepatic GHR. As expected, the insulin-induced phosphorylation of IR, Akt (S473), FoxO1 and GSK3β was reduced in both the GAS and Ing of AAV-GHR mice compared with that in AAV-GFP mice (Figure 4M-N), presenting impaired insulin signaling. Collectively, our above results suggested that hepatic GHR overexpression facilitated insulin resistance in skeletal muscle and white adipose tissue, which might be relevant to impaired systemic insulin signaling.

### Hepatic GHR overexpression results in elevation of serum RBP4 levels

**Figure 5:**
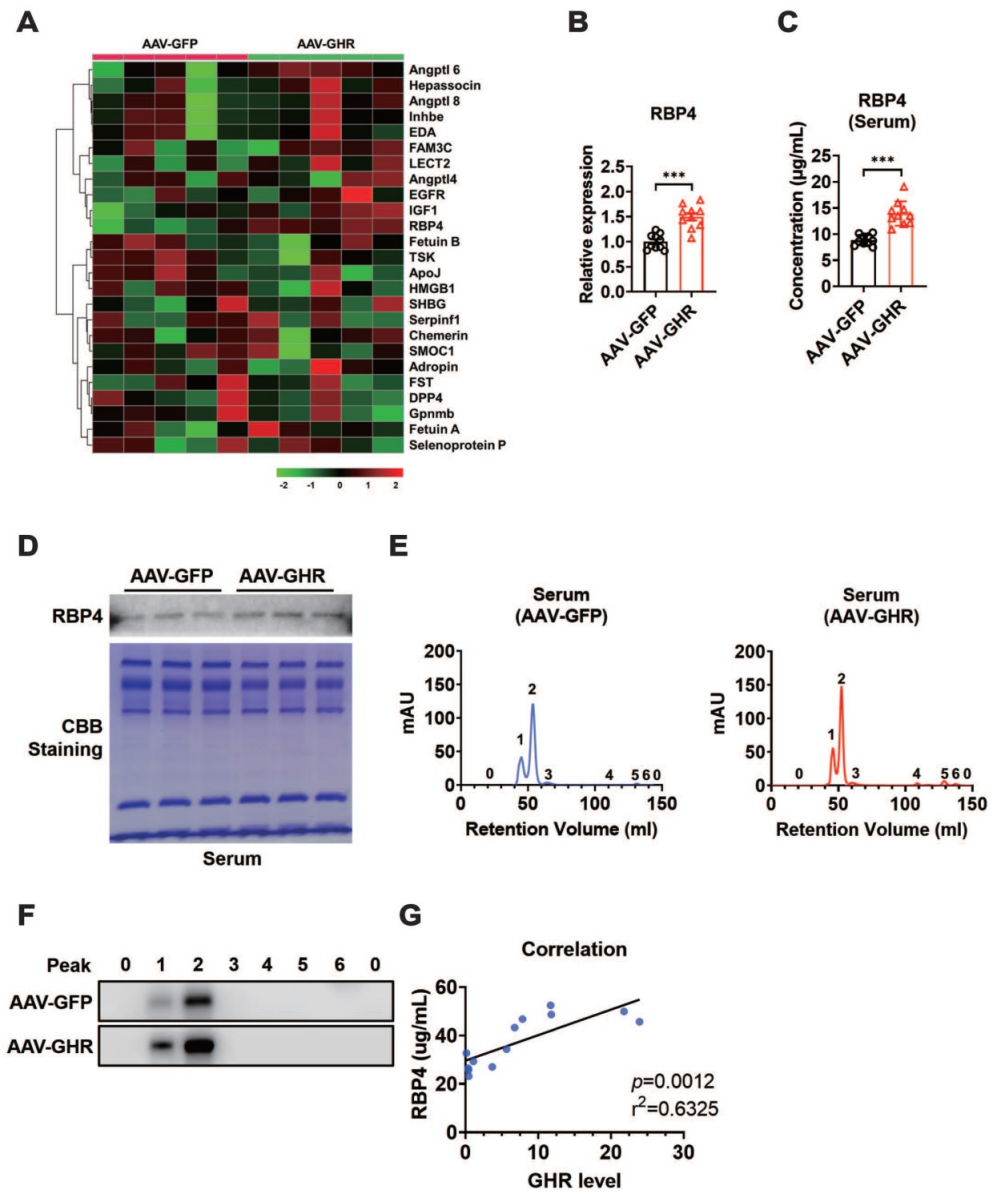
** Hepatic GHR overexpression results in elevation of serum RBP4 levels.** (A) The mRNA levels of hepatokines were determined by qRT-PCR in the livers of AAV-infected mice, which were plotted in a heatmap (n=5). (B) Relative mRNA levels of RBP4 in the livers of AAV-infected mice (n=10). (C) The concentrations of serum RBP4 of AAV-infected mice (n=10). (D) Western blot and SDS-PAGE analysis were performed in the serum of AAV-infected mice. (E) Elution profile of chylomicrons in the serum of AAV-GFP (left) or AAV-GHR (right) mice. Purified proteins were detected in column eluents by monitoring absorbance at 280 nm. (F) Western blots of RBP4 in serum of AAV-GFP (up) or AAV-GHR (down) mice, which were separated by gel filtration chromatography and collected according to the ultraviolet absorption peak of fractions. (G) The correlation between serum RBP4 concentrations and hepatic GHR levels in humans (n=13). Data are expressed as the mean ± SD. ***p < 0.001 (Student's* t*-test).

Impaired insulin signaling was present in the liver, skeletal muscle and white adipose tissue of AAV-GHR mice. Interestingly, GHR overexpression was observed only in the livers of AAV-GHR mice. The most likely explanation is that the abnormal metabolic phenotypes were modulated through interorgan communication mediated by the secretion of hepatokines 1. Therefore, hepatokines, associated with insulin sensitivity, were screened from the NCBI website database PubMed (https://pubmed.ncbi.nlm.nih.gov/) with the search terms “hepatokine” and “insulin sensitivity”, and potential hepatokines were further assessed based on the distributions of these hepatokines using online Gene Portal Systerm-BioGPS (http://biogps.org/). Potential hepatokines are presented, and their mRNA levels were also determined by qRT-PCR in the livers of AAV-infected mice, which were plotted in a heatmap (Figure 5A). Partial least squares-discriminant analysis (PLS-DA) of hepatokines was performed, and their coefficients were calculated (Figure S5A), which provided information about the significance of each variable on the latent variables. Based on the above analysis, we speculated that hepatokine-RBP4 might contribute more to insulin sensitivity after AAV-GHR injection (Figure S5A), the PLS-DA coefficient of which was the highest. The histogram analysis of RBP4 is also shown in Figure 5B, which observably revealed an increased RBP4 mRNA level in the liver of AAV-GHR mice. Then, the serum level of RBP4 was determined to verify our hypothesis. Compared with AAV-GFP mice, the serum concentrations of RBP4 were obviously elevated in AAV-GHR mice (Figure 5C). In addition, the hepatic mRNA levels of RBP4 and the serum concentrations of RBP4 in GHR-KO mice were also measured to further confirm our conjecture. Contrary to the above data, both hepatic RBP4 mRNA levels and serum RBP4 concentrations were suppressed in GHR-KO mice compared to GHR-WT mice (Figure S5B-C). Next, SDS-PAGE and Western blot analysis were also performed to examine the protein level of RBP4 in the serum of AAV-GHR mice. As shown in Figure 5D, Coomassie brilliant blue (CBB) staining was used for protein correction, and the expression of RBP4 was upregulated in the serum of AAV-GHR mice, which was consistent with hepatic RBP4 expression, while the protein level of RBP4 was decreased in the liver of GHR-KO mice (Figure S5D). After purification by the ÄKTA purifier system, the different protein fractions contained in serum were collected for Western blot analysis according to the ultraviolet absorption peak (Figure 5E). The protein fractions of RBP4 were gathered in “Peak 1” and “Peak 2”. Additionally, RBP4 protein overexpression was observed in AAV-GHR mice compared to AAV-GFP mice (Figure 5E-F). In summary, evidence obtained from multiple lines revealed that hepatic GHR overexpression induced the elevation of serum RBP4 levels. The opposite results observed in the serum of GHR-KO mice further supported the above hypothesis (Figure S5E-F). In addition, increased RBP4 levels were also present in the serum and liver of obese subjects compared with control subjects (Figure S5G-H). Moreover, the correlations between the serum RBP4 levels and the hepatic GHR mRNA levels were analyzed in human specimens (Figure 5G), further suggesting an association between hepatic GHR and circulating RBP4.

### GHR activates RBP4 transcription by activating STAT5

**Figure 6:**
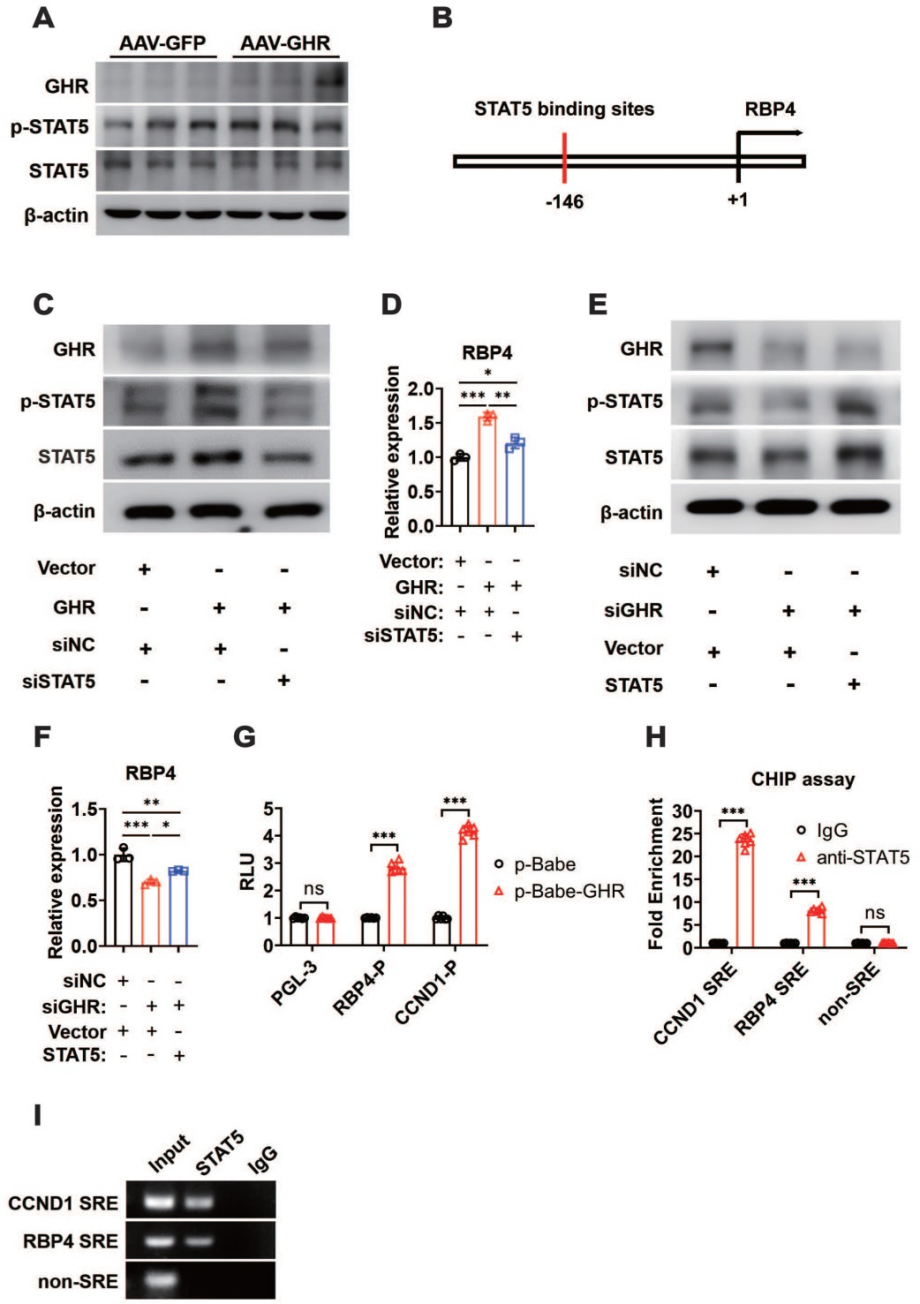
** GHR activates RBP4 transcription by activating STAT5.** (A) Western blots analysis of GHR, p-SAT5 and STAT5 in the livers of AAV-infected mice. (B) The schematic representation of STAT5 binding site in the promoter region of RBP4. (C) Western blots analysis of GHR, p-SAT5 and STAT5 in HepG2 cells transfected with GHR plasmid and/or siSTAT5 as indicated. (D) Relative mRNA level of RBP4 in the HepG2 cells transfected with GHR plasmid and/or siSTAT5 as indicated. (E) Western blots analysis of GHR, p-SAT5 and STAT5 in HepG2 cells transfected with siGHR and/or STAT5 plasmid as indicated. (F) Relative mRNA level of RBP4 in the HepG2 cells transfected with siGHR and/or STAT5 plasmid as indicated. (G) The HepG2 cells were cotransfected with different plasmids, then the luciferase activity was determined after 48 h-transfection (n=6). (H and I) ChIP assay was performed by using anti-STAT5 antibody. The elutes were analyzed by using primers for CCND1 STAT5 response elements (SRE), RBP4 SRE, or non-SRE region. The quantitative results were obtained by real-time PCR (H, n=6) or electrophoretic assay (I). Data are expressed as the mean ± SD. ns, no significant, *p < 0.05, **p < 0.01, ***p < 0.001 (Student's* t*-test or one-way ANOVA).

GHR usually transduces signals by phosphorylating downstream targets, including JAK2 and STAT pathway factors. Moreover, GHR signals as a class Ⅰ cytokine receptor via STAT5, the phosphorylation of which regulates target gene transcription by binding to responsive elements in the promoter region of its target gene 20, 38. As expected, the phosphorylation of STAT5 was enhanced when GHR was overexpressed in the livers of AAV-GHR mice (Figure 6A), while it was suppressed in the livers of GHR-KO mice (Figure S6A). The potential STAT5 binding site was identified within the promoter region of RBP4, which was conserved across species (Figure 6B, S6B). The relationship between GHR/STAT5 and RBP4 was determined *in vitro*. Increased phosphorylation of STAT5 was observed in HepG2 cells transfected with the GHR expression plasmid, which was accompanied by an elevated mRNA level of RBP4, while the elevated RBP4 level was reversed by the transfection of small interfering STAT5 (siSTAT5) (Figure 6C-D). In contrast, the phosphorylation of STAT5 was reduced in HepG2 cells transfected with small interfering GHR (siGHR), which was accompanied by a decreased mRNA level of RBP4, while the attenuated RBP4 level could be reversed by the transfection of STAT5 expression plasmid (Figure 6E-F), suggesting that GHR activates RBP4 transcription by activating STAT5. Moreover, luciferase reporter assays were also performed. An RBP4 luciferase reporter vector containing a STAT5 responsive element (SRE) was generated and the promoter activity was also evaluated in HepG2 cells after cotransfection for 48 h. A cyclin D1 (CCND1) reporter was constructed and used as a positive control 39, 40. As shown in Figure 6G, the transfection of p-Babe-GHR into HepG2 cells activated the activity of the RBP4 promoter and the CCND1 promoter. However, the activity of the RBP4 promoter containing SRE was inhibited in HepG2 cells transfected with siGHR oligos (Figure S6C), indicating that the SRE site could contribute to the regulation by STAT5. To determine whether STAT5 could be recruited to the promoter region of RBP4, a ChIP assay was performed in AAV-GHR mice. Consistent with the data from the luciferase reporter assay, STAT5 could be recruited to SRE in the RBP4 promoter (Figure 6H-I). Taken together, our data demonstrated that RBP4 was a downstream gene of GHR and that GHR facilitated the expression of RBP4 via the phosphorylation activation of STAT5.

### GHR promotes RBP4 protein homeostasis through the HIF1α/TTR axis

**Figure 7:**
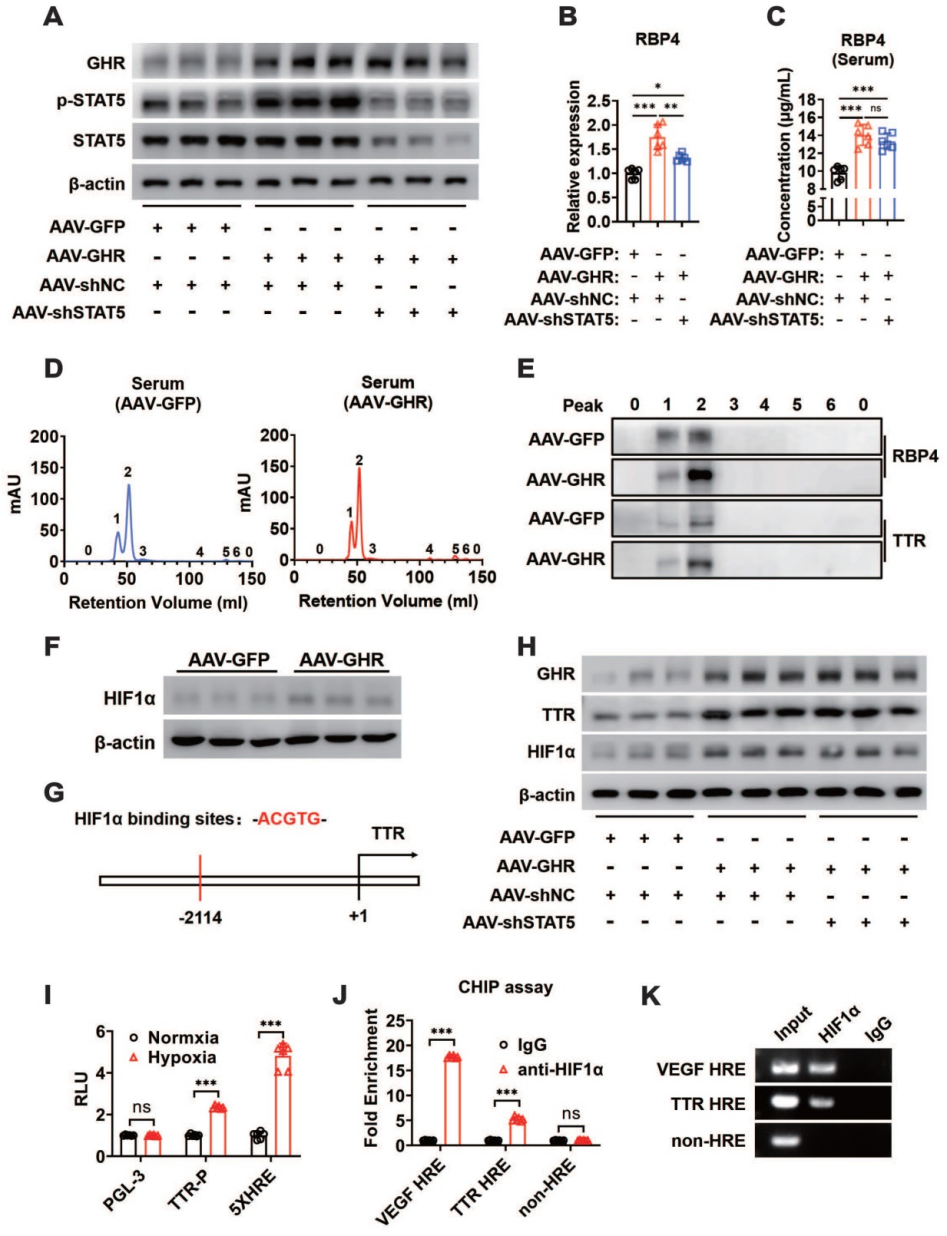
** GHR promotes RBP4 protein homeostasis through the HIF1α/TTR axis.** (A) Western blots analysis of GHR, p-SAT5 and STAT5 in the livers of AAV-infected mice as indicated. (B) Relative mRNA levels of RBP4 in the livers of AAV-infected mice as indicated (n=6). (C) The concentrations of serum RBP4 of AAV-infected mice as indicated (n=6). (D) Elution profile of chylomicrons in the serum of AAV-GFP (left) or AAV-GHR (right) mice. Purified proteins were detected in column eluents by monitoring absorbance at 280 nm. (E) Western blots of RBP4 and TTR in serum of AAV-GFP or AAV-GHR mice, which were separated by gel filtration chromatography and collected according to the ultraviolet absorption peak of fractions. (F) Western blots analysis of HIF1α in the livers of AAV-infected mice as indicated. (G) The schematic representation of the HIF1α binding site in the promoter region of TTR. (H) Western blots analysis of GHR, TTR and HIF1α in the livers of AAV-infected mice as indicated. (I) The HepG2 cells were transfected with pGL-3 or TTR promoter reporter plasmid or 5x HIF1α response elements (HRE) reporter. After transfection for 24 h, the cells were exposed to a hypoxic condition for 24 h. Then the luciferase activity was determined (n=6). (J and K) ChIP assay was performed by using anti-HIF1α antibody. The elutes were analyzed by using primers for VEGF HRE, TTR HRE, or non-HRE region. The quantitative results were obtained by real-time PCR (J, n=6) or electrophoretic assay (K). Data are expressed as the mean ± SD. ns, no significant, *p < 0.05, **p < 0.01, ***p < 0.001 (Student's* t*-test or one-way ANOVA).

The mechanism by which GHR activated RBP4 depending on STAT5 was also verified in *in vivo* experiments. The mice were administered AAV-GHR and/or AAV-mediated short hairpin STAT5 (AAV-shSTAT5) by lateral tail vein injection. Western blot analysis revealed that the protein expression of hepatic GHR was increased because of AAV-GHR interference accompanied by the enhanced phosphorylation of STAT5 (Figure 7A). As expected, an overexpressed mRNA level of RBP4 was also detected in AAV-GHR mice, while the mRNA level of RBP4 was reversed after AAV-shSTAT5 interference (Figure 7B). The serum concentrations of RBP4 were further determined to examine whether circulating RBP4 was modified. Beyond our expectation, however, the elevated serum RBP4 level in hepatic GHR overexpression mice could not be attenuated by STAT5 repression (Figure 7C), suggesting that the level of RBP4 secreted by the liver was reduced, but a high steady-state concentration of RBP4 was observed in circulation. The serum protein complexes formed by the combination of RBP4 and TTR resist glomerular filtration and reduce renal clearance of RBP4, as reported in previous studies 18, 19. We hypothesized that the steady state of circulating RBP4 might be modified. Therefore, after purification by the ÄKTA purifier system, the serum protein levels of RBP4 and TTR were reevaluated in AAV-GFP or AAV-GHR mice. Accompanied by serum increased RBP4 protein levels, which was consistent with previous data mentioned above, the protein level of TTR was indeed reinforced in the serum of AAV-GHR mice compared with the protein level of TTR in AAV-GFP mice (Figure 7D-E).

GHR could upregulate HIF1α through the PI3K/Akt pathway as described previously, and heightened HIF1α protein expression was also observed in the livers of AAV-GHR mice (Figure 7F). Furthermore, the potential HIF1α binding site (hypoxic response element, HRE) was identified within the promoter region of TTR (Figure 7G), which suggests that GHR might facilitate the expression of TTR by the activation of HIF1α, resulting in the protein homeostasis of circulating RBP4. The additional loss-of-function experiment laterally verified our conjecture, which presented the results that the protein expression of HIF1α was abated in the liver of GHR-KO mice (Figure S7A), which was accompanied by attenuated serum protein complexes formed by RBP4 and TTR in the serum of GHR-KO mice (Figure S7B-C). Additionally, *in vivo* experiments further proved that hepatic GHR overexpression promoted the protein expression of TTR and HIF1α, which were independent of STAT5 (Figure 7H). In addition, luciferase reporter assays were also used to validate our hypothesis. A TTR luciferase reporter containing potential HRE was generated and the promoter activity was evaluated in HepG2 cells under hypoxic conditions. Hypoxic conditions increased TTR promoter activity (Figure 7I). A reporter containing five repeated HIF1α responsive elements (5x HRE) was used as a positive control 41. Transfection of HIF1α-DM (P402A/P564A) plasmids into HepG2 cells also activated the TTR promoter, suggesting that the HRE site is functional (Figure S7D). To determine whether HIF1α could be recruited to the promoter region of TTR, a ChIP assay was performed in AAV-GHR mice. VEGF HRE was used as a positive control 42. Consistent with the data from the luciferase reporter assay, HIF1α could be directly recruited to the HRE site in both the promoters of TTR and VEGF (Figure 7J-K). Taken together, our data demonstrated that TTR was a direct target gene of HIF1α and that GHR promoted RBP4 protein homeostasis through the HIF1α/TTR axis.

## Discussion

Dysregulation induced by systemic insulin resistance culminates in and sustains the pathophysiological alterations found in metabolic syndrome. Therefore, exploring the potential regulatory mechanism of insulin signaling is helpful to better understand the pathogenesis of systemic insulin resistance. Previous studies have shown that insulin sensitivity was improved in both GHR deficient mice and individuals 43-46. However, whether hepatic GHR overexpression regulates insulin sensitivity remains unknown, and a systematic understanding of how GHR regulates insulin signaling has not been closely examined. In the present study, a mouse model of hepatic GHR overexpression was established to confirm the novel mechanism by which GHR regulates insulin sensitivity by promoting circulating RBP4 and maintaining its stability, which is supported by multiple lines of evidence. First, hepatic GHR is upregulated in multiple obese mouse models and obese humans. Second, hepatic GHR overexpression facilitated the shifts in fuel selection preference to fatty acid oxidation instead of glucose oxidation and enhanced hepatic glucose output. Third, GHR promotes circulating RBP4 levels by provoking the phosphorylation of STAT5 and propels the stability of circulating RBP4 by activating the HIF1α/TTR axis to repress renal clearance of RBP4. Consequently, hepatic GHR overexpression induces systemic insulin resistance. Therefore, our findings identified a new regulatory mechanism by which GHR regulated systemic insulin resistance mediated by hepatokine RBP4.

As proteins secreted by hepatocytes, hepatokines function systemically through interorganizational crosstalk to modulate metabolic syndrome and might serve as potential therapeutic targets for metabolic diseases 1. In our experiment, systemic insulin resistance was observed in the liver, skeletal muscle and white adipose tissue, which might be induced by hepatic GHR overexpression; hence, we presume that hepatokines could be responsible for this effect. Then, RBP4 is screened from a variety of hepatokines and functions systemically through circulation. Circulating RBP4 has previously been reported to be derived from adipocytes 12, 13; however, recent studies proved that hepatocytes are the principal source of circulating RBP4 in mice and transgenic expression of RBP4 in adipose tissue fails to elevate circulating RBP4 levels 11, 15. Increased circulating RBP4 levels were also observed in hepatic GHR overexpression mice, while they were reduced in hepatic GHR deficiency mice, indicating that RBP4 secretion was mediated by hepatic GHR. GHR typically acts by initiating a signal transduction cascade through the classic JAK5/STAT5 pathway. Interestingly, the STAT5 binding site was identified within the upstream region of RBP4, enhanced phosphorylation of STAT5, activated by overexpressed GHR, and promoted RBP4 release into the circulation. In addition, this study also proved that RBP4 was one of the downstream targets of STAT5.

Increasingly, elevated circulating RBP4 in both obesity and diabetes is generally believed to have double effects. On the one hand, in the process of glucose metabolism, circulating RBP4 could reduce insulin sensitivity by stimulating Glut4 translocation and decreasing glucose uptake 12, 47, which was also revealed in our study. Moreover, the activation of RBP4 accelerates gluconeogenesis to expedite the synthesis and output of hepatic glucose, which would also provoke the secretion of insulin to maintain glucose homeostasis 48, 49. Consequently, insulin signaling is impaired and insulin resistance occurs, which would also be compensated for by increased insulin secretion 49. In addition, as previously reported, RBP4 transcription could be regulated by a multiprotein complex and RBP4 expression may be regulated as part of a network of pathways relevant to the onset of T2D 50. On the other hand, in the process of lipid metabolism, circulating RBP4 stimulated basal lipolysis and impaired insulin inhibition of lipolysis in white adipose tissue and enhanced the utilization of fatty acids in skeletal muscle, which would also facilitate insulin resistance 15, 47, 51. Thus, hepatic GHR overexpression induced systemic insulin resistance through intertissue crosstalk mediated by circulating RBP4.

Based on the above study, we finally investigated whether RBP4 could recover to its baseline level since STAT5 was silenced *in vivo*. As expected, the RBP4 levels were elevated both in the liver and serum with the injection of AAV-GHR. Hepatic RBP4 levels were reversed after the AAV-shSTAT5 intervention. However, the serum RBP4 level did not return to baseline, which was also promoted despite shSTAT5 involvement. The different levels of RBP4, observed in serum and liver inspired us to look for the reasons for these levels. Therefore, the stability of circulating RBP4 must be considered, as reported in a previous study 18. As a binding partner for RBP4, TTR reduced the glomerular filtration rate of RBP4 and retained it in the blood 52. Thus, TTR binding for RBP4 is a crucial determinant for the stability of circulating RBP4. However, the expression of hepatic TTR was overexpressed whether shSTAT5 was disturbed, which means that an unknown signaling pathway regulating the stability of circulating RBP4 was activated, independent of STAT5. We must explore other regulatory pathways of GHR. As a downstream target of GHR, the PI3K/Akt/mTOR pathway can be provoked by GHR through JAK2 20. Consequently, multiple regulators were stimulated. HIF1α is expressed under the control of growth factor signaling, particularly the PI3K/Akt/mTOR pathway 53-55. In our present experiment, the HIF1α binding site was identified within the upstream region of TTR by sequence alignment. We also proved that TTR was the downstream target of HIF1α. Moreover, our current study revealed that circulating RBP4 homeostasis was improved through the HIF1α/TTR axis mediated by GHR, resulting in lessened renal clearance of RBP4 and enhanced circulating RBP4. Furthermore, the PI3K/Akt pathway could drive malignant transformation when chronically activated 56, which might also aggravate insulin resistance. Together, this evidence provides important insights into systemic insulin resistance mediated by hepatic GHR.

Taken together, our current study demonstrated the functional importance of hepatic GHR in the regulation of glucose homeostasis, which was mediated by circulating RBP4. The present data proved that GHR not only promoted the secretion of RBP4 from the liver by activating STAT5 but also maintained circulating RBP4 homeostasis and repressed renal clearance of RBP4 by provoking activation of the HIF1α/TTR axis, thus inducing systemic insulin resistance. Our findings shed light on the novel mechanism by which GHR regulates insulin signaling pathways, which could provide a potential target for glucose homeostasis.

## Supplementary Material

Supplementary figures and tables.

## Abbreviations

Angptl4: angiopoietin-like protein 4
Angptl6: angiopoietin-like protein 6
Angptl8: angiopoietin-like protein 8
ApoJ: apolipoprotein J
ATGL: adipose triglyceride lipase
DPP4: dipeptidyl peptidase 4
EDA: ectodysplasin-A
EGFR: epidermal growth factor receptor
FAM3C: family with sequence similarity 3: member C
FoxO1: forkhead box protein O1
FST: follistatin
G6Pase: glucose-6-phosphatase
GFP: green fluorescent protein
Glut4: glucose transporter 4
Gpnmb: glycoprotein non-metastatic melanoma protein B
HIF1α: hypoxia-inducible factor-1
HMGB1: high mobility group box 1
HSL: hormone-sensitive lipase
IGF-1: insulin-like growth factor-1
Inhbe: inhibin βE
LECT2: leukocyte cell-derived chemotaxin 2
PDH: pyruvate dehydrogenase
PDK4: pyruvate dehydrogenase kinase isozyme 4
PEPCK: phosphoenolpyruvate carboxykinase
PGC1α: peroxisome proliferative activated receptor gamma coactivator 1α
PLIN5: perilipin 5
Serpinf1: serpin family F member 1
SHBG: sex hormone-binding globulin
SMOC1: SPARC related modular calcium binding 1
TSK: tsukushi
VEGF: vascular endothelial growth factor
