# Supplementary material for: Growth hormone receptor disrupts glucose homeostasis via promoting and stabilizing retinol binding protein 4
Source: Theranostics. 2021 Jul 13;11(17):8283–300. doi: 10.7150/thno.61192 (PMC8344001; doi:10.7150/thno.61192)

Supplementary materials

Table S1. Characteristics of human subjects

	**Control** (n=6)	**Obesity** (n=7)
Age (year)	47.33±12.19	43.00±11.52
Body weight (kg)	57.83±12.59	118.27±19.93***
Height (cm)	165.67±8.85	167.83±10.23
BMI (kg/m^2)	20.96±3.41	41.84±4.89***
Serum TG (mM)	1.03±0.30	2.49±0.42***
Serum GH (ng/mL)	12.21±1.59	9.94±1.75*
Serum IGF-1 (ng/mL)	377.36±43.26	356.93±33.91
Serum Insulin (μU/mL)	6.23±0.97	15.79±1.77***

Data were expressed as mean ± SD. *$p < 0.05$, ***$p < 0.001$, versus control group (Student's *t-test*).

Table S2. Primer sequences for ChIP

ID	Sense Sequence (5' → 3')	Antisense Sequence (5' → 3')
non-SRE	CTGACTATTTCGATCAGGCT	TTTCTTCTATGAGACGCACC
CCND1-SRE	CTCAACGAAGCCAATCAAGA	AATCGCTGCAAAGTTATTAGTCG
RBP4-SRE	TAAAAATGCATGGTAAACACTTGGC	TGGTGCTGTTTGGGTCAATATTTAT
non-HRE	GTTGGTTTCTAAGGCTGATG	AAGACCAGGCTAACCTTGA
VEGF-HRE	CGAGGGTTGGCGGCAGGAC	CAGTGGCGGGGAGTGAGACG
TTR-HRE	AGAGTGAGTTCCAGGACAGC	TTACATAAGGATGTCCCCTGAT

SRE, STAT5 response elements; HRE, HIF1α response elements.

Table S3. Primer sequences for siRNA

ID	Sense Sequence (5’ → 3’)	Antisense Sequence (5’ → 3’)
N.C.	UUCUCCGAACGUGUCACGU	ACGUGACACGUUCGGAGAA
siGHR	ACAUAAUCAGGGCAUUCUUUCCAtt	UGGAAAGAAUGCCCUGAUUAUGUtt
siSTAT5	UGAUGUUGAACAGUUUCUGUGCCtt	GGCACAGAAACUGUUCAACAUCAtt

Table S4. Primer sequences for qRT-PCR

Gene	Sense Sequence (5' → 3')	Antisense Sequence (5' → 3')
*m*GHR	CTGCAAAGAATCAATCCAAGCC	CAGTTCAGGGGAACGACACTT
*m*G6Pase	CGACTCGCTATCTCCAAGTGA	GTTGAACCAGTCTCCGACCA
*m*PEPCK	CTGCATAACGGTCTGGACTTC	CAGCAACTGCCCGTACTCC
*m*PGC1α	CAATGAATGCAGCGGTCTTA	GTGTGAGGAGGGTCATCGTT
*m*PLIN5	CAGAGCAAACACCGTACCCAG	GGGATGGAAAGTAGGGCTAGG
*m*FoxO1	TCAAGGATAAGGGCGACAGC	TGTCCATGGACGCAGCTCTT
*m*PDK4	AGGGAGGTCGAGCTGTTCTC	GGAGTGTTCACTAAGCGGTCA
*m*Glut4	GTGACTGGAACACTGGTCCTA	CCAGCCACGTTGCATTGTAG
*m*ATGL	CAACGCCACTCACATCTACGG	GGACACCTCAATAATGTTGGCAC
*m*HSL	TCCCTCAGTATCTAGGCCAGA	GGCTCATTTGGGAGACTTTGTTT
*m*RBP4	AGTCAAGGAGAACTTCGACAAGG	CAGAAAACTCAGCGATGATGTTG
*m*Angptl6	CTGGGCCGTCGTGTAGTAG	CAGTCCTCTAGGAGTATCAGCAG
*m*Hepassocin	CCCTGTCAGGAACTTTTCATCC	CGGTAGTAAACACCGTTCAGGT
*m*Angptl8	CCAGCCTGTCGGAGATTCAG	GTGGCTCTGCTTATCAGCTCG
*m*Inhbe	CTAACCAGCCGTCCCAGAATA	GTGCCCGGAAAAGAGGGAG
*m*EDA	GTGGACGGCACCTACTTCATC	CACCATCTTCACGGCGATTT
*m*FAM3C	GGACTCAGCCATTCGTTCTAC	GCTGCTCCACTAGCCATCTTAAA
*m*LECT2	CCCACAACAATCCTCATTTCAGC	ACACCTGGGTGATGCCTTTG
m Angptl4	CATCCTGGGACGAGATGAACT	TGACAAGCGTTACCACAGGC
*m*EGFR	GCATCATGGGAGAGAACAACA	CTGCCATTGAACGTACCCAGA
*m*IGF1	AAATCAGCAGCCTTCCAACTC	GCACTTCCTCTACTTGTGTTCTT
*m*Fetuin B	TGCCAAGGTTCTACGGTCCA	CAGCAGGGTTCTCATCTCCAG
*m*TSK	TGCAGGGCATCCTCCATCTA	GCCTGAAAACACCTCAGCTC
*m*ApoJ	ACAATGGCATGGTCCTGGGAGAG	GTATGCTTCAGGCAGGGCTTGC
*m*HMGB1	GCTGACAAGGCTCGTTATGAA	CCTTTGATTTTGGGGCGGTA
*m*SHBG	TCTGCTGTTGCTACTACTGATGC	GGGCCATTGCTGAGGTACTTA
*m*Serpinf1	GCCCTGGTGCTACTCCTCT	CGGATCTCAGGCGGTACAG
*m*Chemerin	GCTGATCTCCCTAGCCCTATG	CCAATCACACCACTAACCACTTC
*m*SMOC1	AATCCACAGGCTACTGTTGGT	CATCGGCCTCTATGCTCTTGG
*m*Adropin	CTCATCGCCATCGTCTGCAAT	GGGACTGGATTCCGAGAGAGA
*m*FST	TGCTGCTACTCTGCCAGTTC	GTGCTGCAACACTCTTCCTTG

*m*DPP4	ACCGTGGAAGGTTCTTCTGG	CACAAAGAGTAGGACTTGACCC
*m*Gpnmb	AGAAATGGAGCTTTGTCTACGTC	CTTCGAGATGGGAATGTATGCC
*m*Fetuin A	ATCCGCTCCACAAGGTACAG	GGTCCAAAGCATGGCAAGT
*m*Selenoprotein P	AGCCATTAAGATCGCTTACTGTG	GAGGGCTCCGCAGTTTTATTG
*m*GAPDH	ACATCATCCCTGCATCCACT	GTCCTCAGTGTAGCCCAAG
*h*GHR	AATGCAGATATTCAGAAAGGAT	ATAATTTCCAGAGTTTCGTTGT
*h*RBP4	GAGTTCTCCGTGGACGAGAC	TCCAGTGGTCATCATTTCCTTTC
*h*GAPDH	ATGGGGAAGGTGAAGGTCG	GGGGTCATTGATGGCAACAATA
18S	ACCGCACTAGGAATAATGGA	CAAATGCTTTCGCTCTGGTC

Figure S1. Fasting blood glucose are elevated in human and different mouse models of metabolic disorder

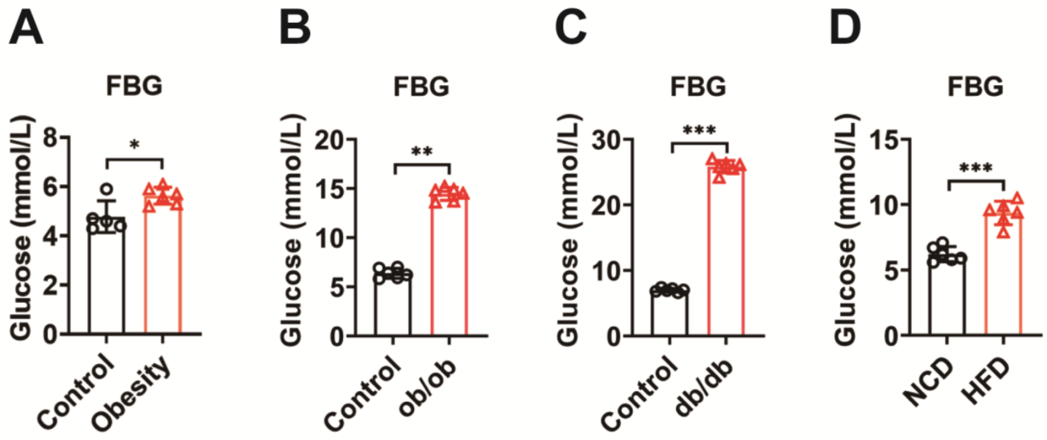

(A) Fasting blood glucose (FBG) levels examined after fasted for 12 h in control and obese humans (n=5-6). (B and C) Fasting blood glucose (FBG) levels examined after fasted for 12 h in *ob/ob* mice (B, n=6) or *db/db* mice (C, n=6), respectively. (D) FBG levels examined after fasted for 12 h in the mice fed with NCD or HFD for 12 weeks (n=6). Data are expressed as the mean ± SD. *$p < 0.05$; **$p < 0.01$; ***$p < 0.001$ (Student's *t*-test).

Figure S2. Body weight and blood glucose levels are reduced in GHR-KO mice

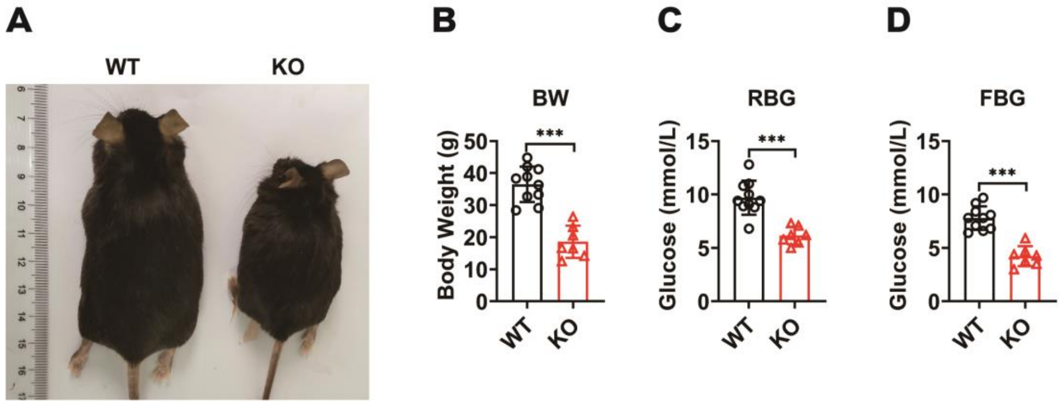

(A and B) Representative photograph (A) and body weight (B, n=7-10) of 12-week-old male GHR$^{-/-}$ (KO) mice, littermate wild-type (WT) mice used as the control group. (C and D) The RBG (C, n=7-10) and FBG (D, n=7-10) levels of GHR-WT or GHR-KO mice. Data are expressed as the mean ± SD. ***$p < 0.001$ (Student's *t*-test).

Figure S3. Hepatic gluconeogenesis and insulin sensitivity are improved in the livers of GHR-KO mice

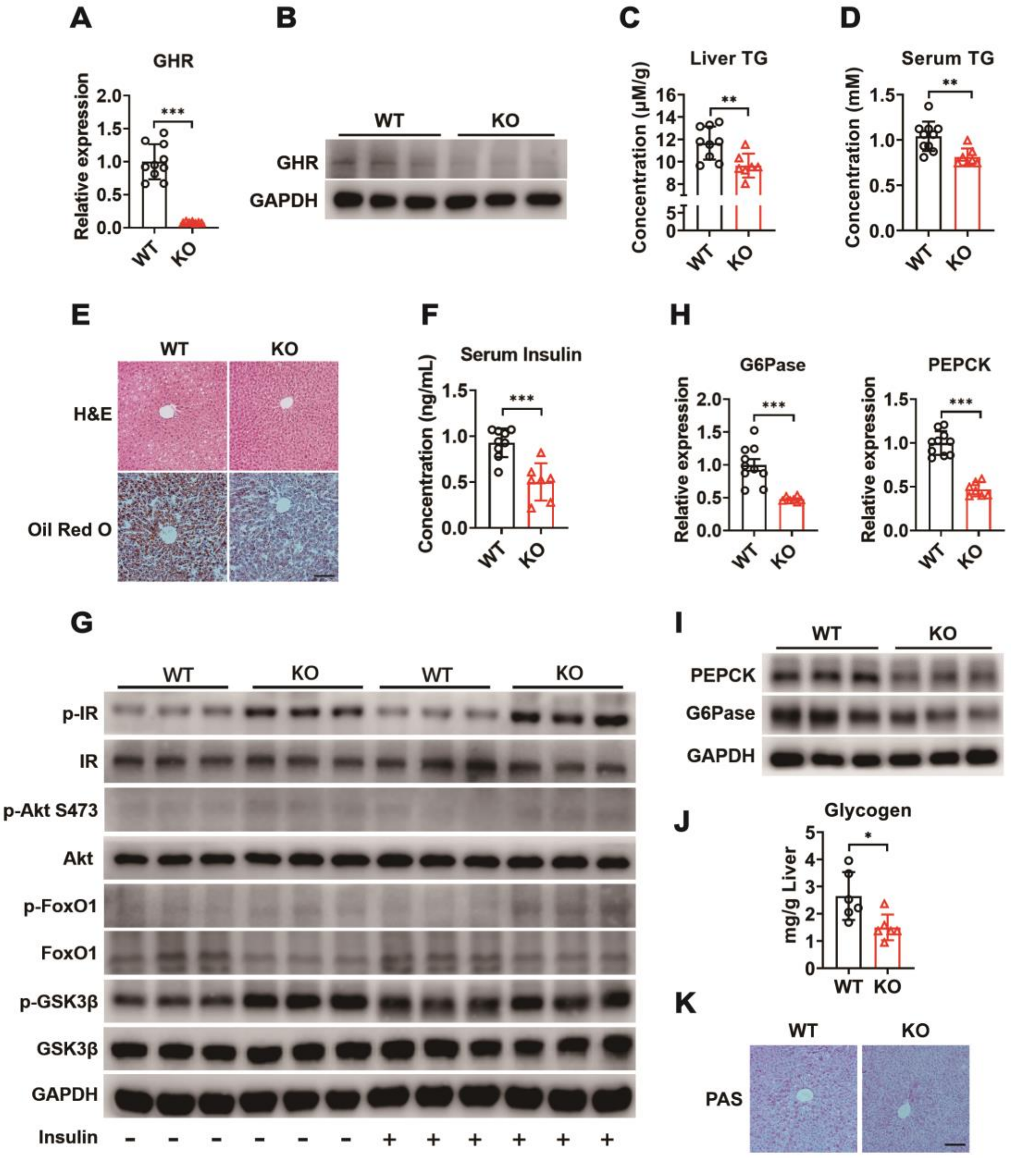

(A and B) Relative mRNA levels (A, n=7-10) and protein levels (B) of GHR in the livers of GHR-WT or GHR-KO mice. (C and D) The TG levels in the livers (C, n=7-9) and serum (D, n=7-10) of GHR-WT or GHR-KO mice. (E) Representative images of H&E staining (up) and Oil Red O staining (down) of liver sections from GHR-WT (left) or GHR-KO (right) mice. Scale bar, 500 μm. (F) The serum insulin levels of GHR-WT or GHR-KO mice (n=7-9). (G) Western blots analysis of phosphorylated key molecules of insulin signaling pathway in the livers of GHR-WT or GHR-KO mice after insulin administration. (H and I): Relative mRNA levels (H, n=7-10) and protein levels (I) of gluconeogenesis-related genes or proteins in the livers of GHR-WT or GHR-KO mice, respectively. (J) The amount of glycogen of GHR-WT or

GHR-KO mice normalized based on liver weight (n=6). (K) Representative images of PAS staining of liver sections from GHR-WT (left) or GHR-KO (right) mice. Scale bar, 500 μm. Data are expressed as the mean ± SD. *$p < 0.05$; **$p < 0.01$; ***$p < 0.001$ (Student's *t*-test).

Figure S4. Hepatic GHR overexpression induces skeletal muscle atrophy and white fat accumulation

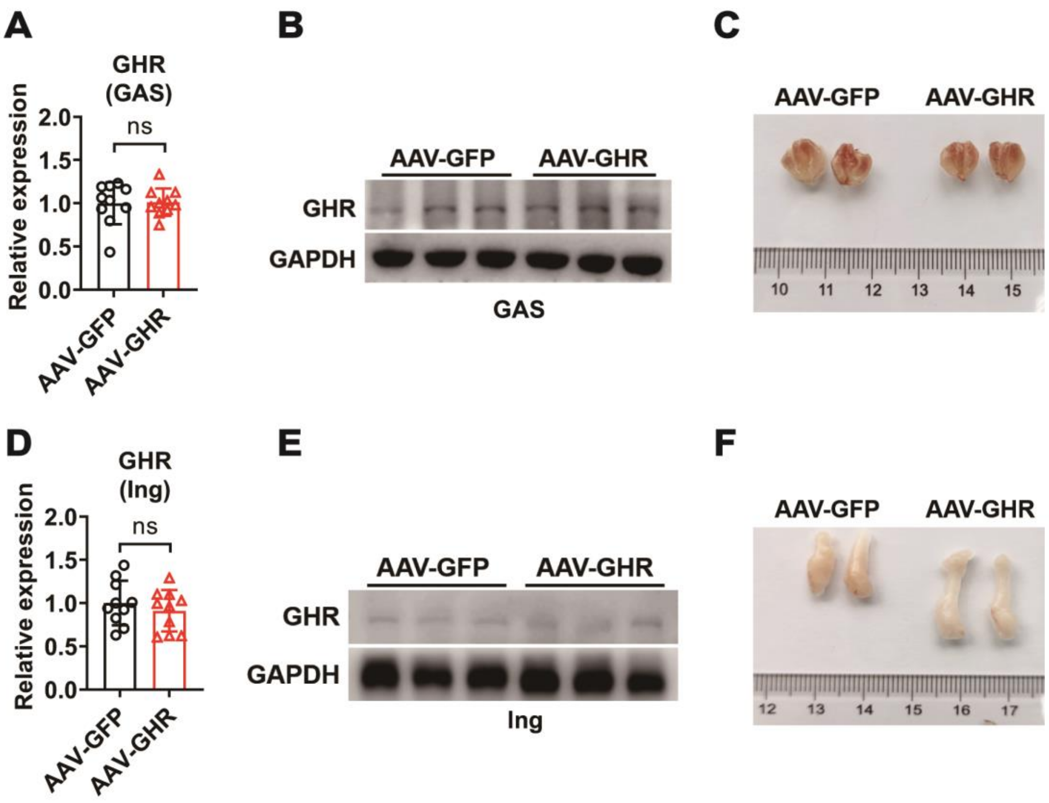

(A and B) Relative mRNA levels (A, n=10) and protein levels (B) of GHR in the GAS of AAV-infected mice. (C) Representative GAS photograph of AAV-infected mice. (D and E) Relative mRNA levels (D, n=10) and protein levels (E) of GHR in the Ing of AAV-infected mice. (F) Representative Ing photograph of AAV-infected mice. Data are expressed as the mean ± SD. ns, no significant (Student's *t*-test).

Figure S5. The level of RBP4 is reduced in the serum of GHR-KO mice

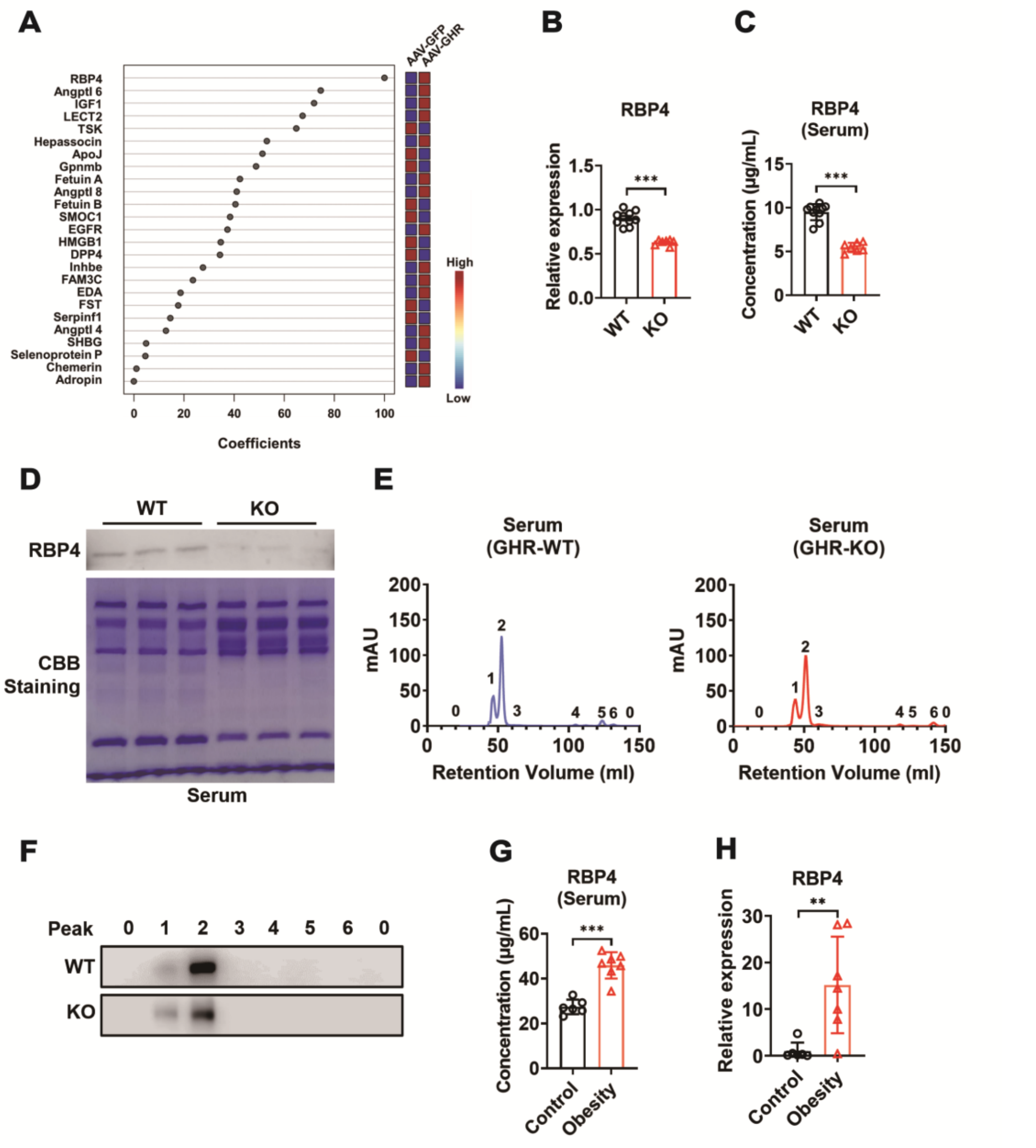

(A) The partial least squares-discriminant analysis (PLS-DA) of hepatokines were performed and the coefficients of them were shown as indicated. (B) Relative mRNA levels of RBP4 in the livers of GHR-WT or GHR-KO mice (n=7-10). (C) The concentrations of serum RBP4 of GHR-WT or GHR-KO mice (n=7-10). (D) Western blot and SDS-PAGE analysis were performed in the serum of GHR-WT or GHR-KO mice. (E) Elution profile of chylomicrons in the serum of GHR-WT (left) or GHR-KO (right) mice. Purified proteins were detected in column eluents by monitoring absorbance at 280 nm. (F) Western blots of RBP4 in serum of GHR-WT (up) or GHR-KO (down) mice, which were separated by gel filtration chromatography and collected according to ultraviolet absorption peak of fractions. (G) The concentrations of serum RBP4 of human (n=6-7). (H) Relative mRNA levels of RBP4 in the livers of

human (n=6-7). Data are expressed as the mean ± SD. $^{**}p < 0.01$, $^{***}p < 0.001$ (Student's *t*-test).

Figure S6. The inhibition of GHR induces depressed transcriptional activity of RBP4

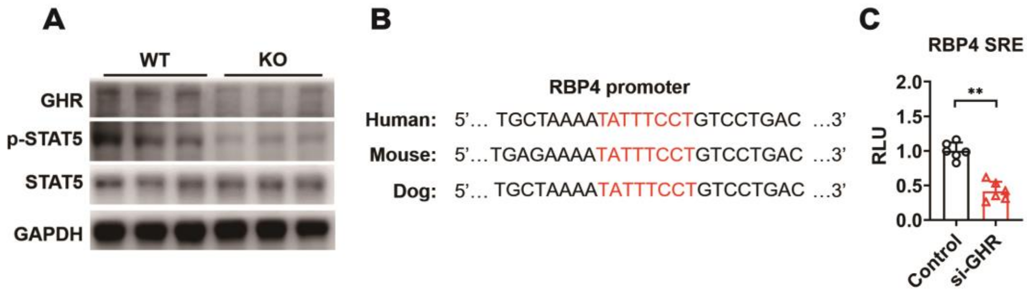

(A) Western blots analysis of GHR, p-SAT5 and STAT5 in the livers of GHR-WT or GHR-KO mice. (B) Sequence alignment of RBP4 promoter from various species. (C) The HepG2 cells were cotransfected with siGHR and RBP4 SRE reporter plasmids. Luciferase activity was analyzed after transfection for 48 h (n=6). Data are expressed as the mean ± SD. $^{**}p < 0.01$ (Student's *t*-test).

Figure S7. The inhibition of HIF1α induces depressed RBP4 expression

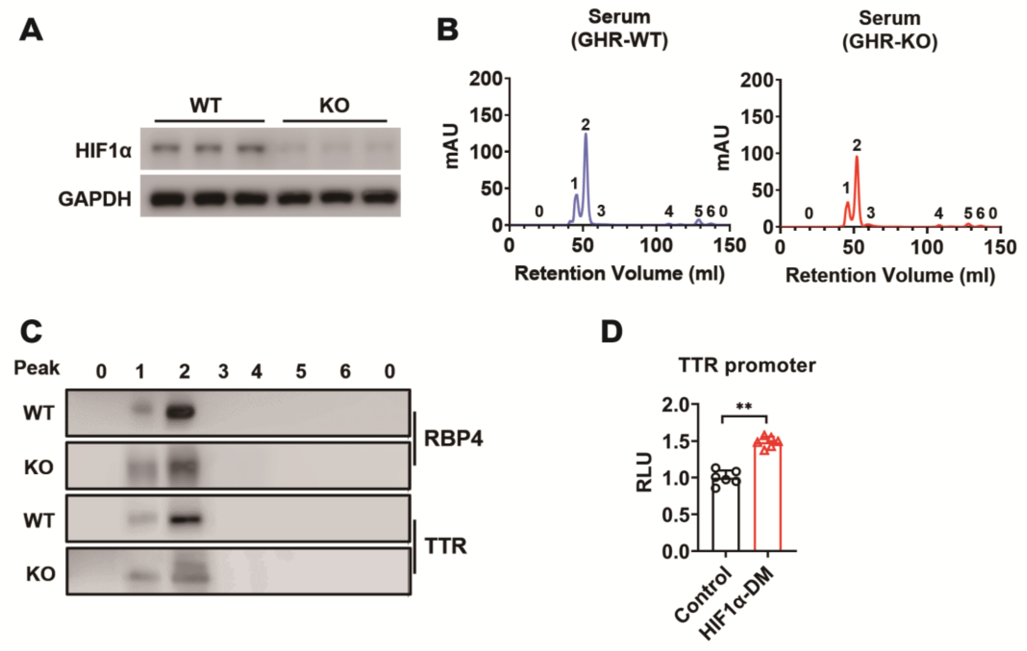

(A) Western blots analysis of HIF1α in the livers of GHT-WT or GHR-KO mice. (B) Elution profile of chylomicrons in the serum of GHR-WT (left) or GHR-KO (right) mice. Purified proteins were detected in column eluents by monitoring absorbance at 280 nm. (C) Western blots of RBP4 and TTR in serum of GHR-WT or GHR-KO mice, which were separated by gel filtration chromatography and collected according to ultraviolet absorption peak of fractions. (D) The HepG2 cells were cotransfected with HIF1α-DM and TTR promoter reporter plasmids. Luciferase activity was analyzed after transfection for 48 h (n=6). Data are expressed as the mean ± SD. $^{**}p < 0.01$ (Student's *t*-test).

Figure S8. Uncropped scans of the Western blots shown in Figures as indicated.

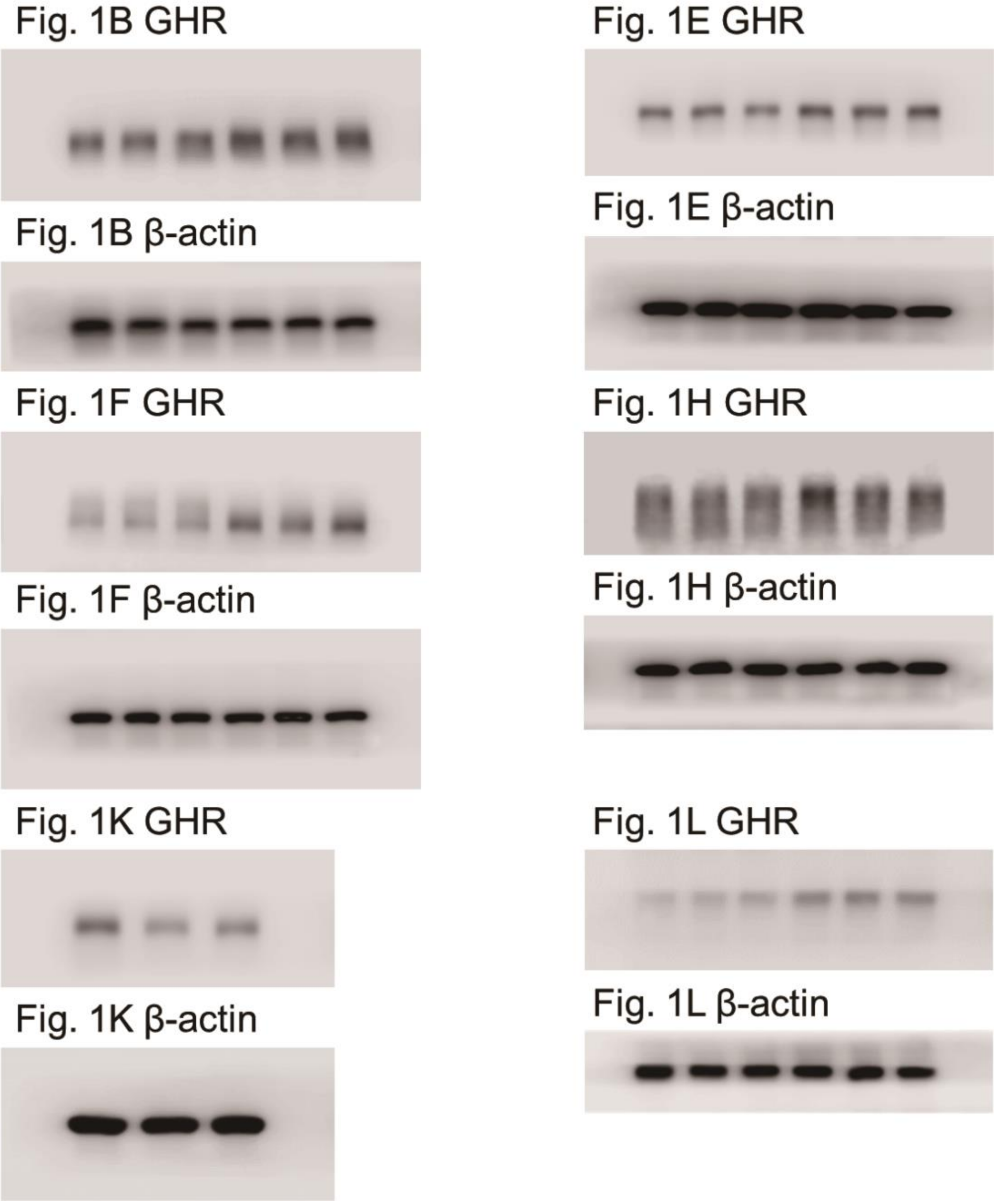

Fig. 3B GHR

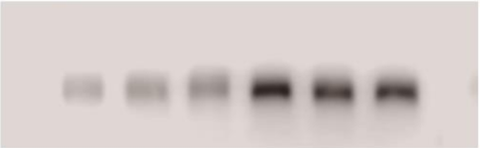

Fig. 3B β-actin

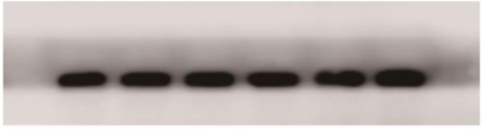

Fig. 3I p-IR

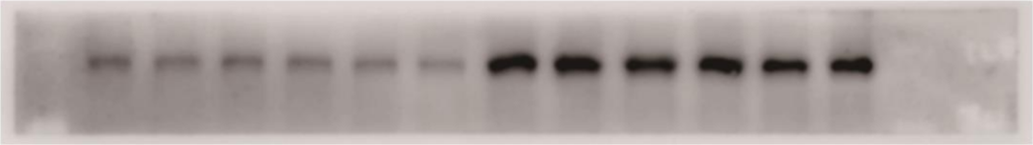

Fig. 3I IR

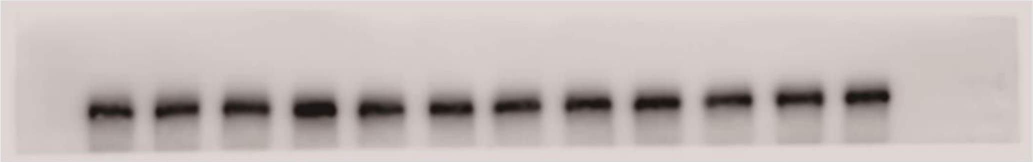

Fig. 3I p-Akt S473

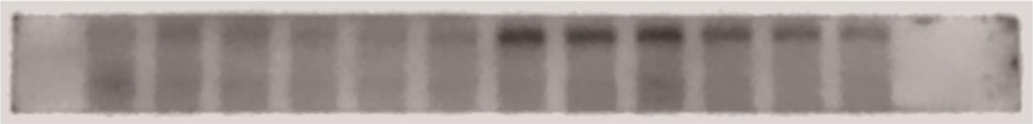

Fig. 3I Akt

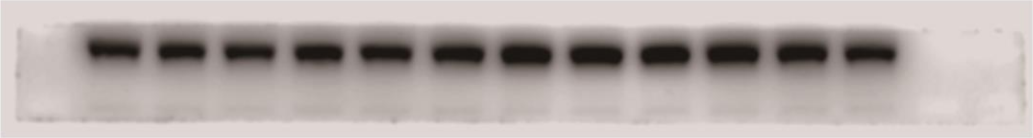

Fig. 3I p-FoxO1

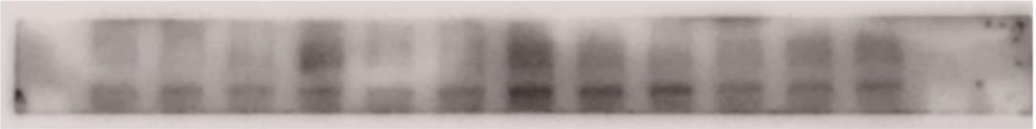

Fig. 3I FoxO1

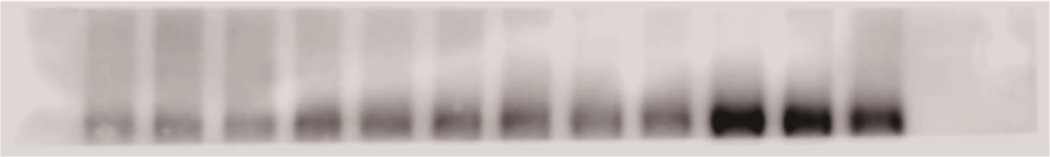

Fig. 3I p-GSK3β

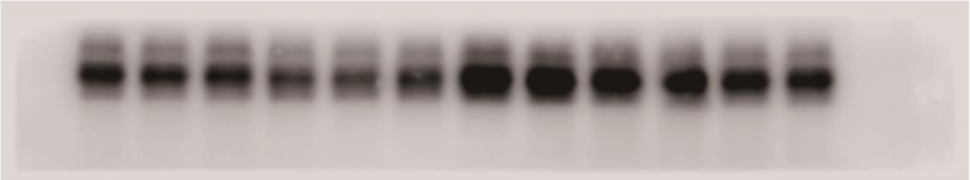

Fig. 3I GSK3β

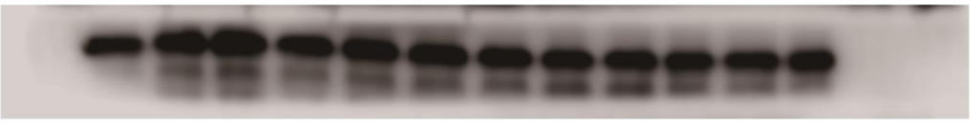

Fig. 3I β-actin

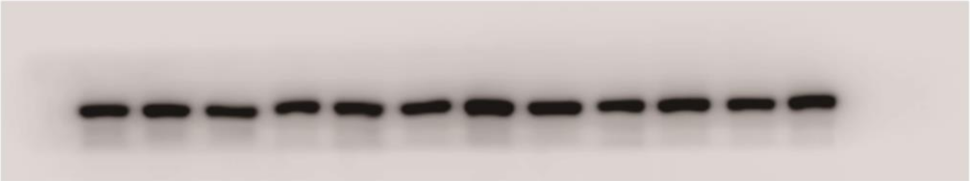

Fig. 3K PEPCK

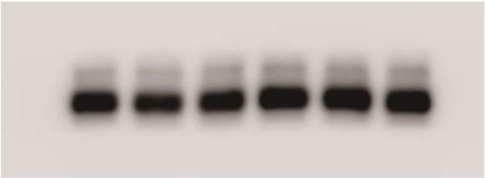

Fig. S3B GHR

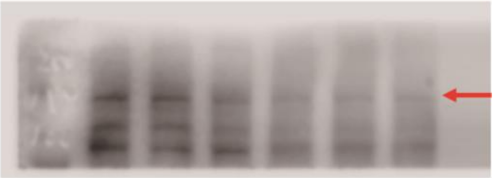

Fig. 3K G6Pase

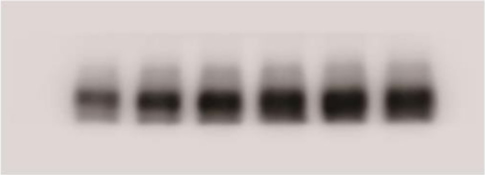

Fig. S3B GAPDH

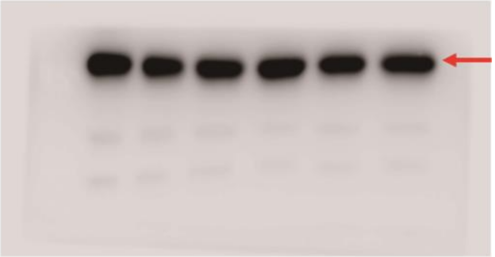

Fig. 3K β-actin

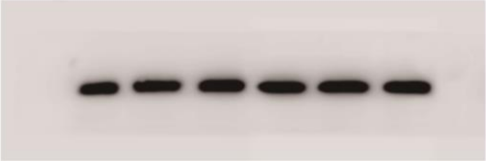

Fig. S3G p-IR

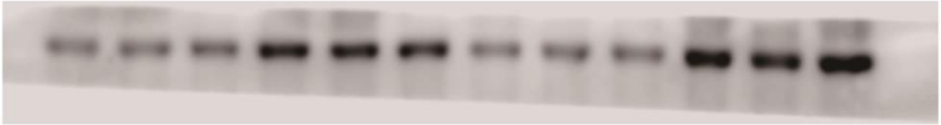

Fig. S3G IR

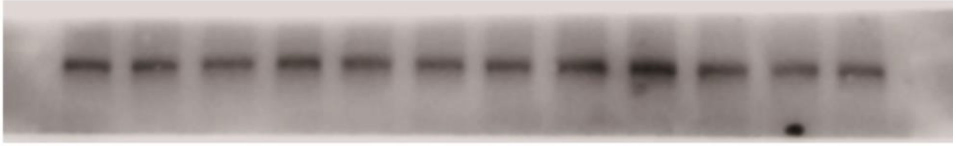

Fig. S3G p-Akt S473

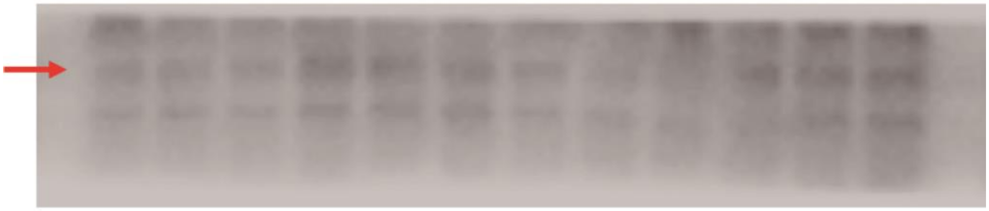

Fig. S3G Akt

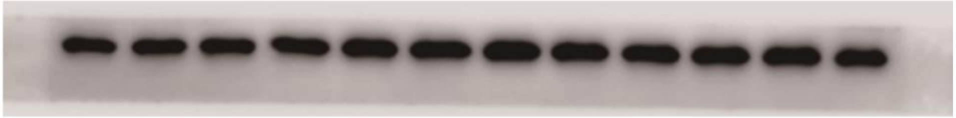

Fig. S3G p-FoxO1

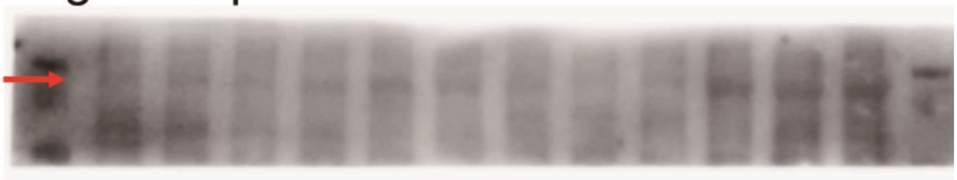

Fig. S3G FoxO1

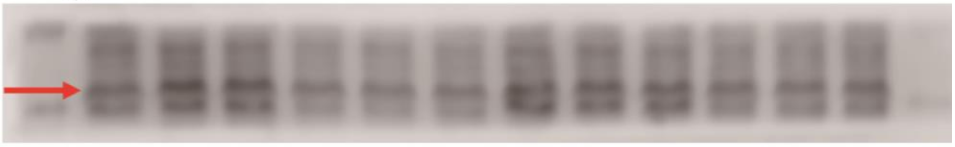

Fig. S3G p-GSK3β

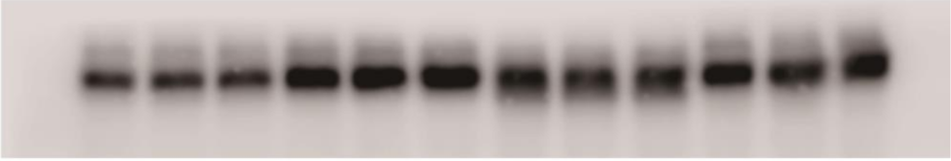

Fig. S3G GSK3β

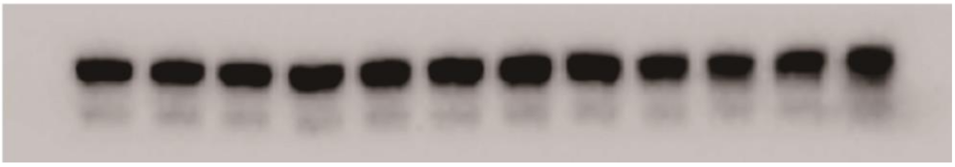

Fig. S3G GAPDH

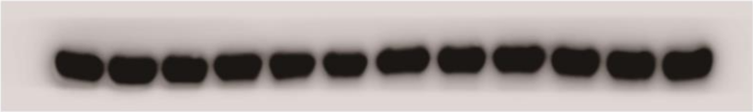

Fig. S3I PEPCK

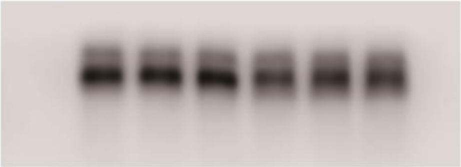

Fig. S3I G6Pase

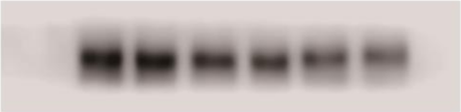

Fig. 4F FoxO1

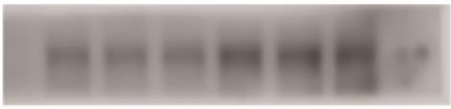

Fig. 4F PDK4

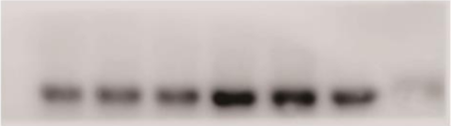

Fig. 4F P-PDH

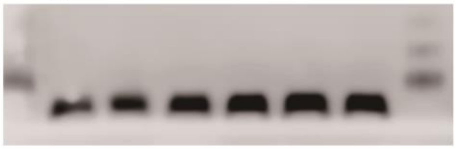

Fig. 4F PDH

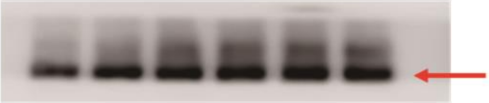

Fig. 4F β-actin

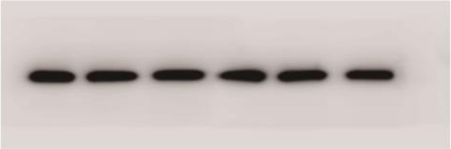

Fig. S3I GAPDH

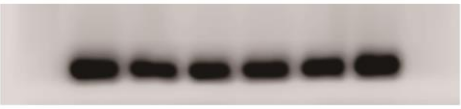

Fig. 4L HSL

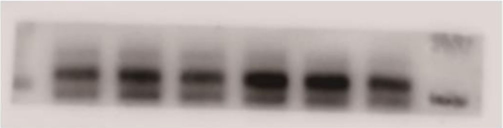

Fig. 4L ATGL

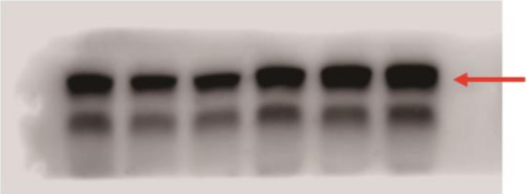

Fig. 4L β-actin

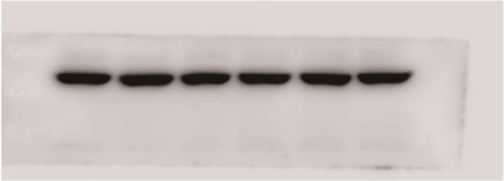

Fig. 4M p-IR

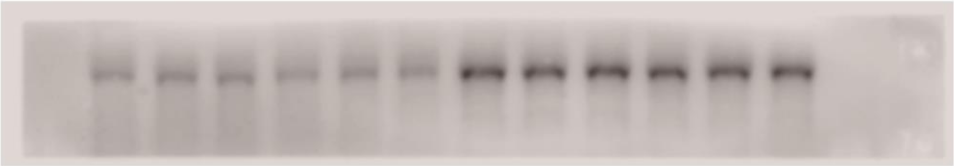

Fig. 4M IR

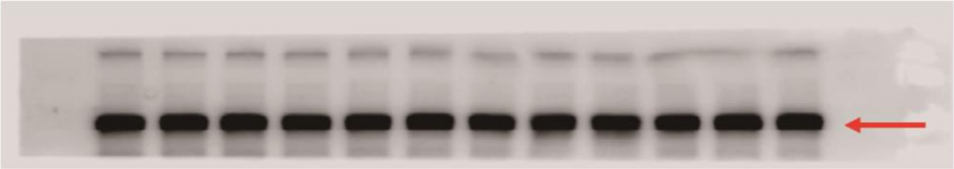

Fig. 4M p-Akt S473

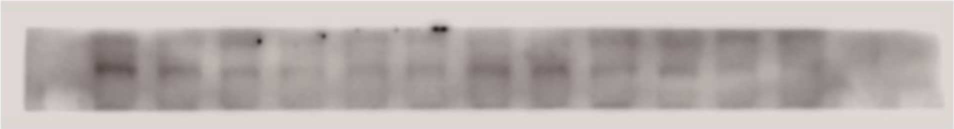

Fig. 4M Akt

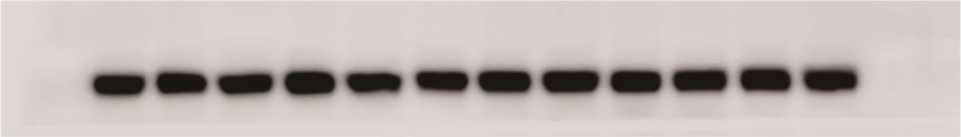

Fig. 4M p-FoxO1

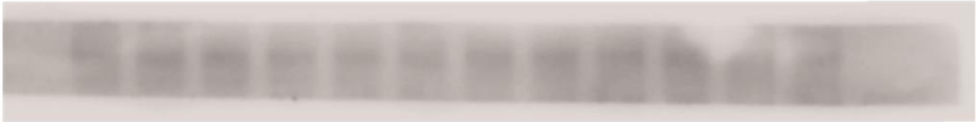

Fig. 4M FoxO1

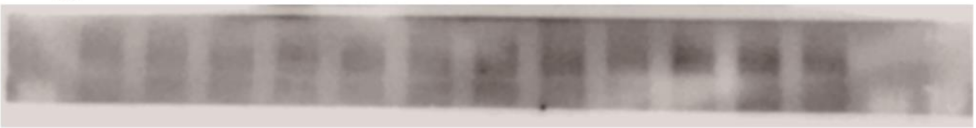

Fig. 4M p-GSK3β

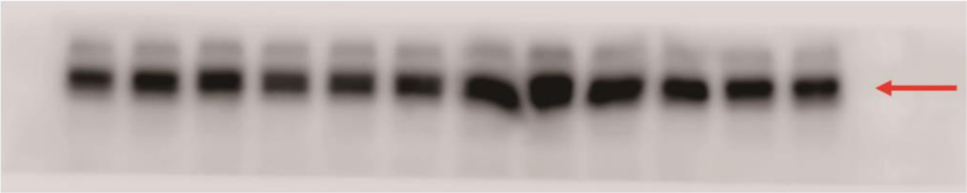

Fig. 4M GSK3β

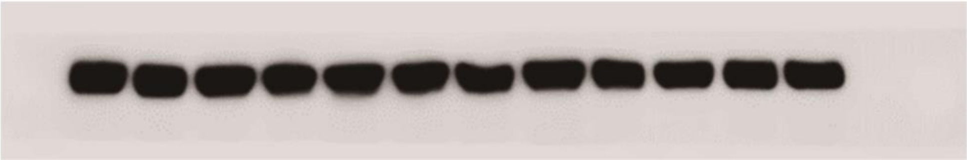

Fig. 4M β-actin

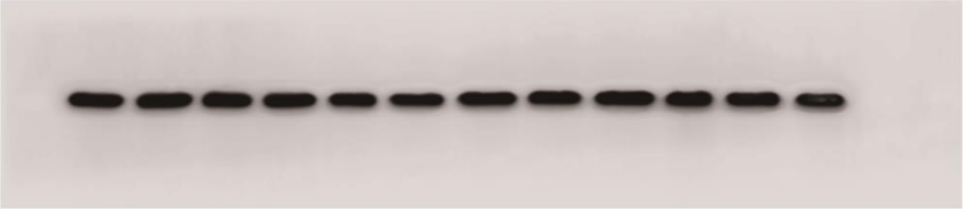

Fig. 4N p-IR

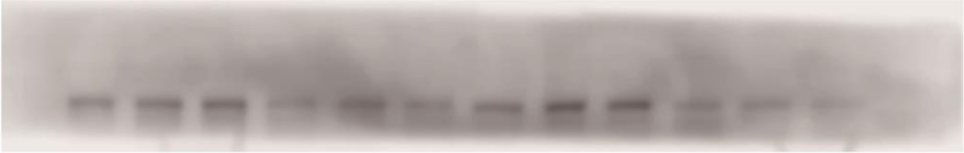

Fig. 4N IR

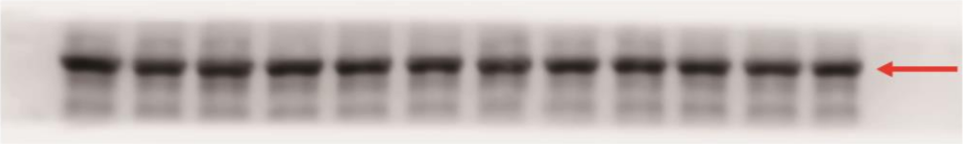

Fig. 4N p-Akt S473

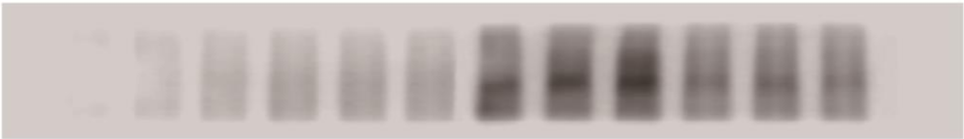

Fig. 4N Akt

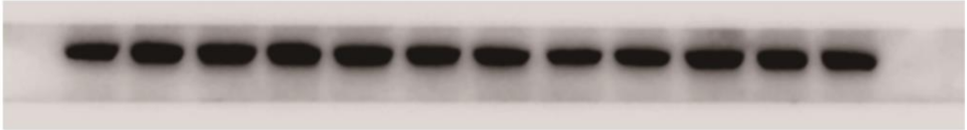

Fig. 4N p-FoxO1

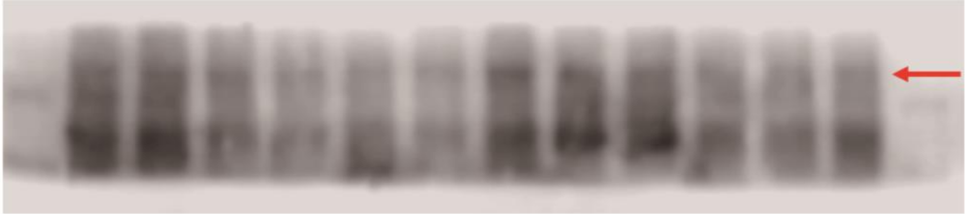

Fig. 4N FoxO1

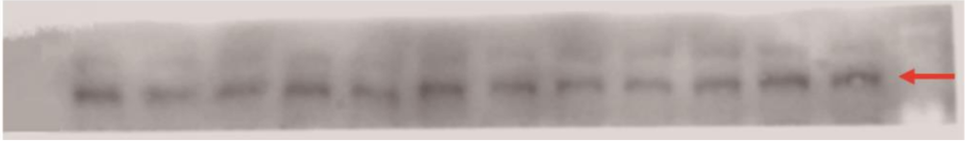

Fig. 4N p-GSK3β

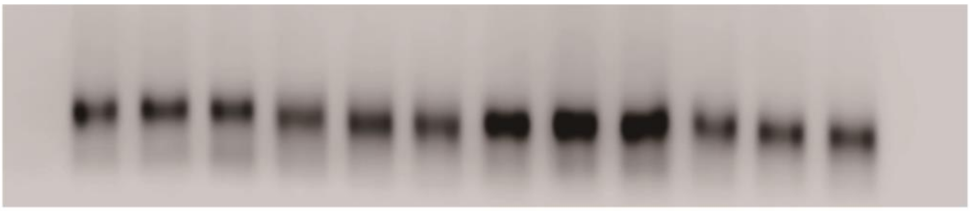

Fig. 4N GSK3β

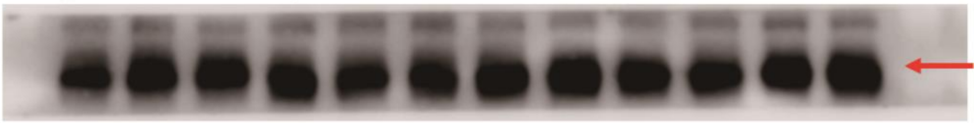

Fig. 4N β-actin

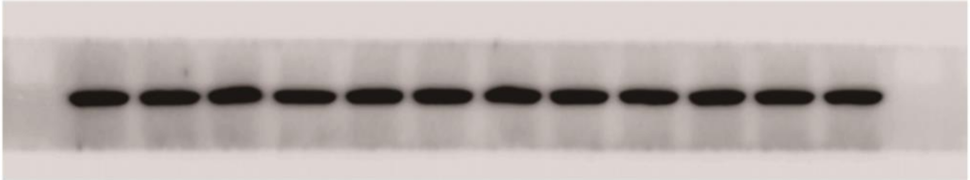

Fig. S4B GHR

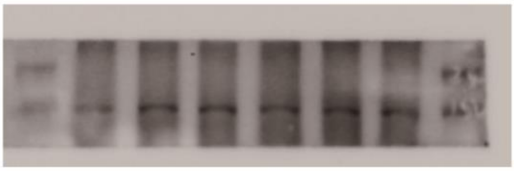

Fig. S4B GAPDH

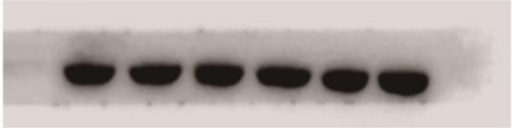

Fig. S4E GHR

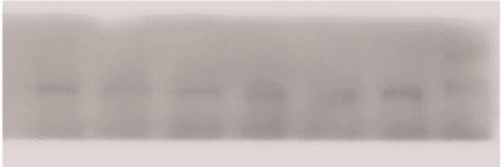

Fig. S4E GAPDH

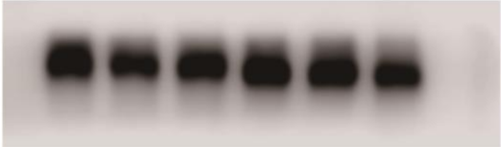

Fig. 5D RBP4

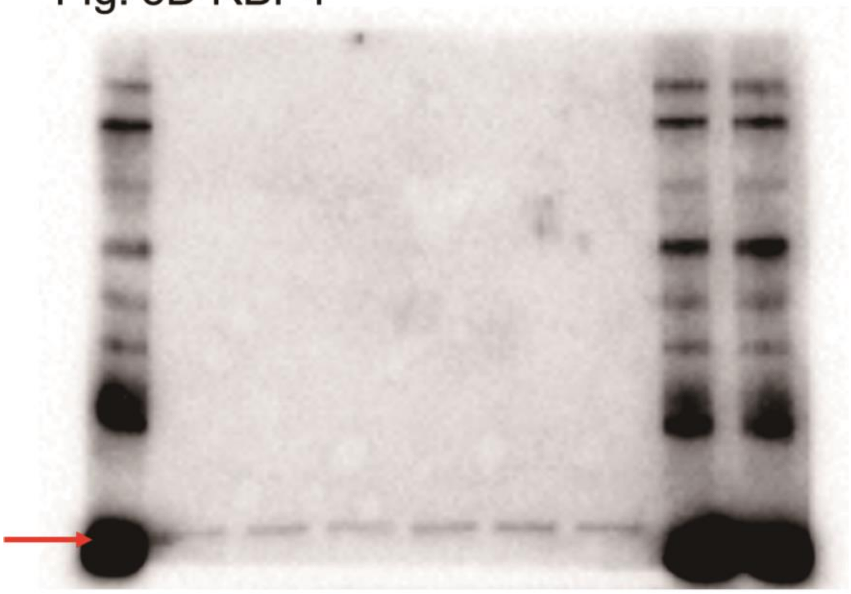

Fig. 5D CBB Staining

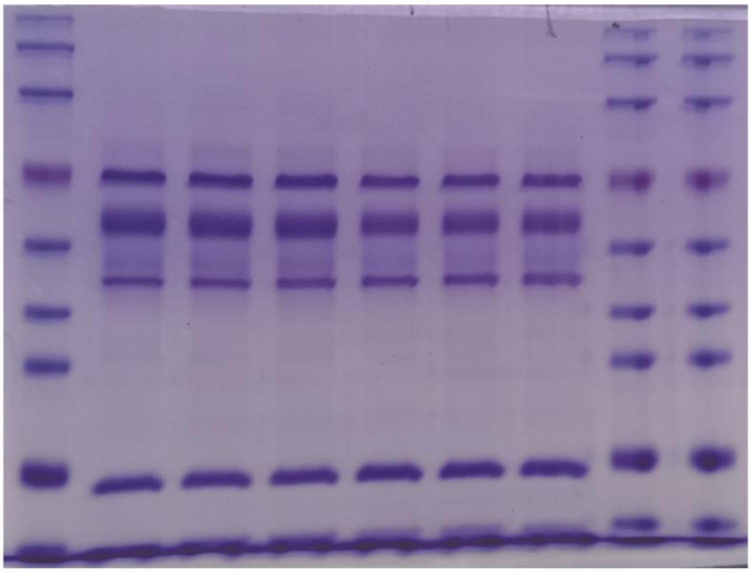

Fig. 5F AAV-GFP

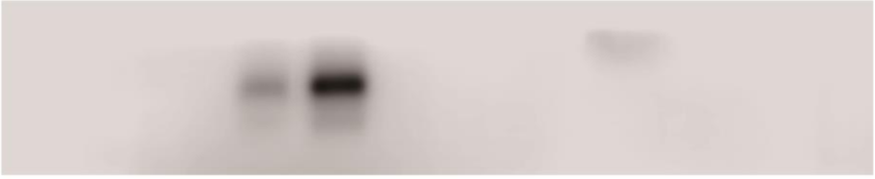

Fig. 5F AAV-GHR

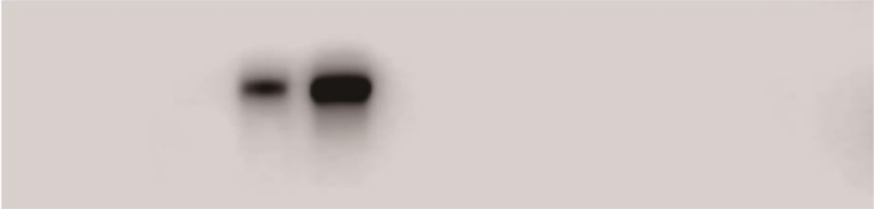

Fig. S5D RBP4

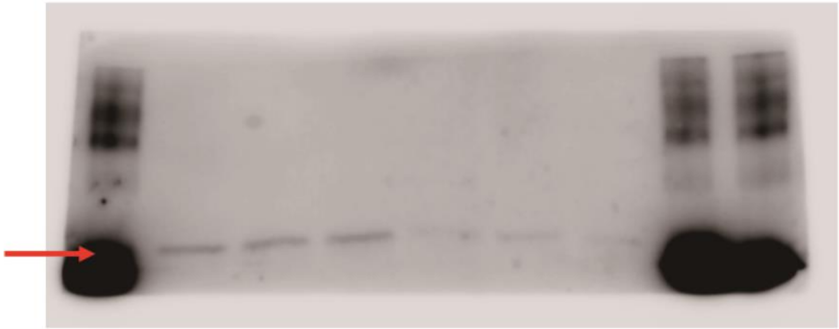

Fig. S5D CBB Staining

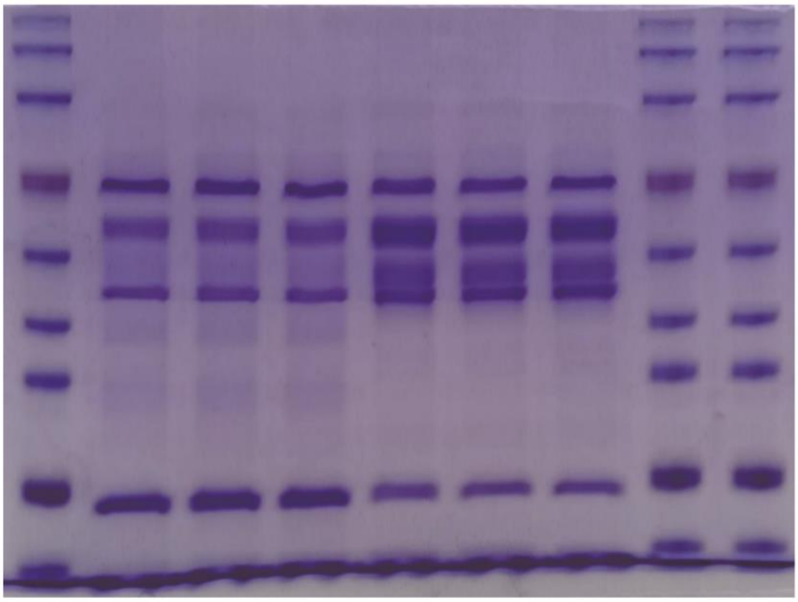

Fig. S5F WT

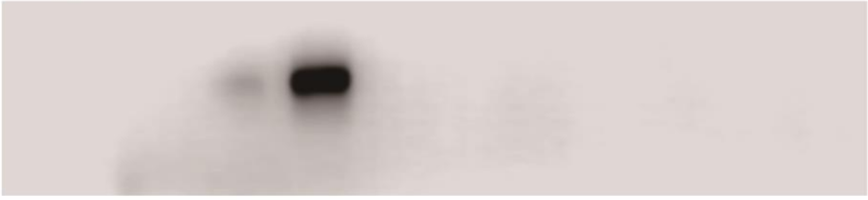

Fig. S5F KO

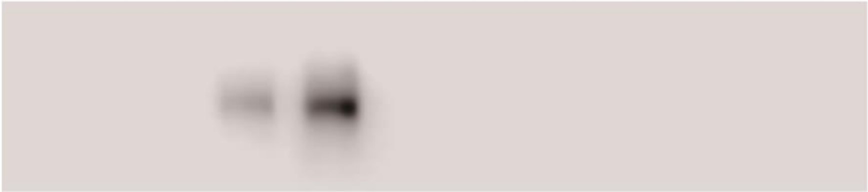

Fig. 6A GHR

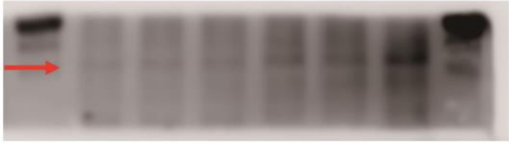

Fig. 6A p-STAT5

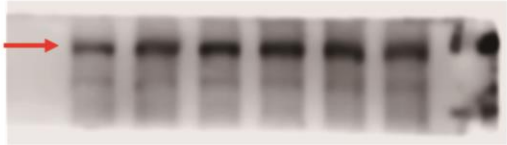

Fig. 6A STAT5

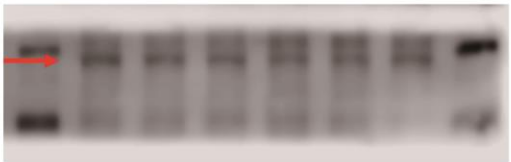

Fig. 6A β-actin

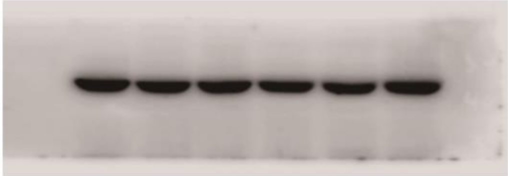

Fig. 6C GHR

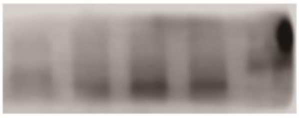

Fig. 6C p-STAT5

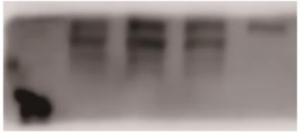

Fig. 6C STAT5

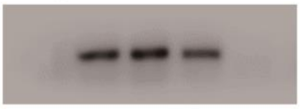

Fig. 6C β-actin

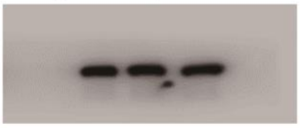

Fig. 6E GHR

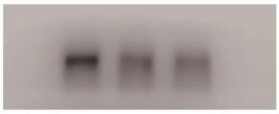

Fig. 6E p-STAT5

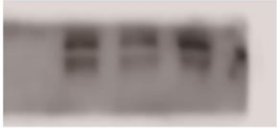

Fig. 6E STAT5

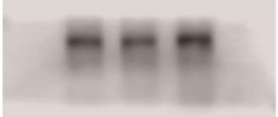

Fig. 6E β-actin

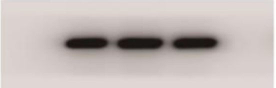

Fig. S6A GHR

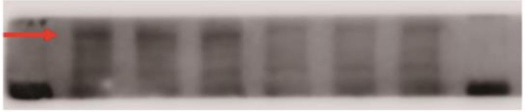

Fig. S6A p-STAT5

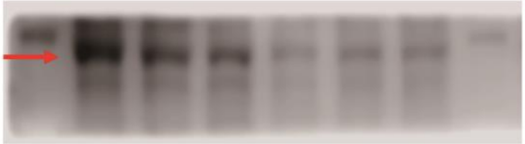

Fig. S6A STAT5

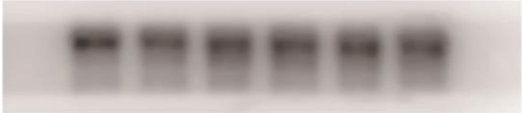

Fig. S6A GAPDH

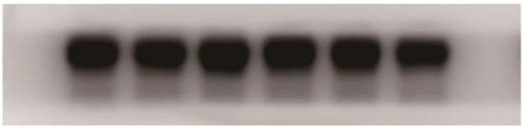

Fig. 7A GHR

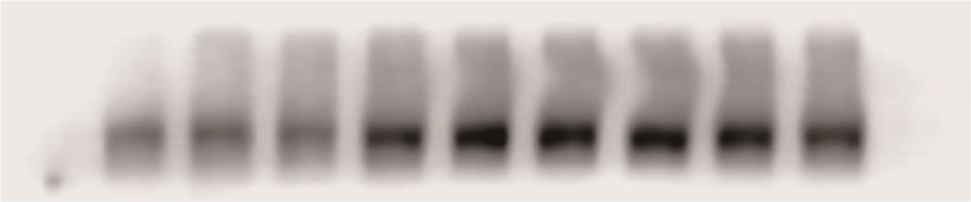

Fig. 7A p-STAT5

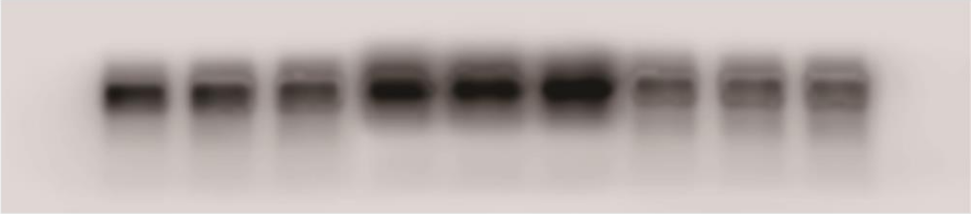

Fig. 7A STAT5

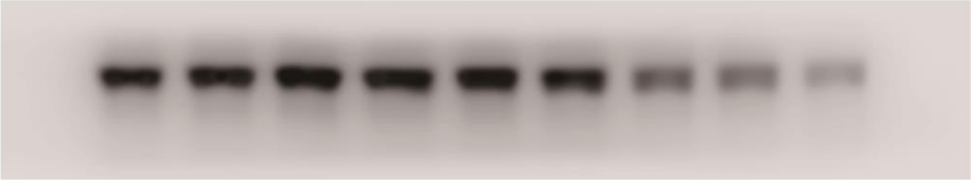

Fig. 7A β-actin

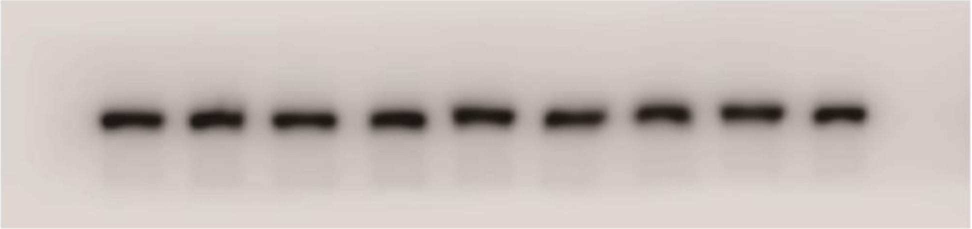

Fig. 7E AAV-GFP-RBP4

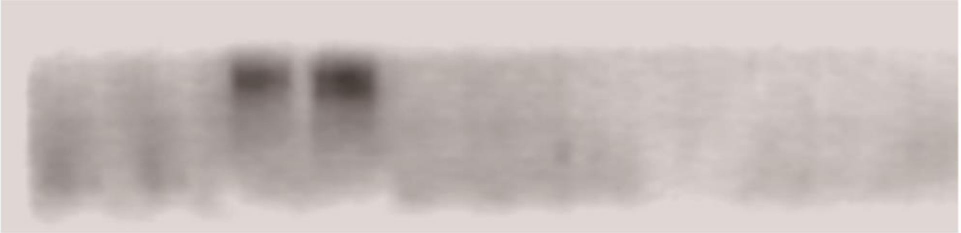

Fig. 7E AAV-GHR-RBP4

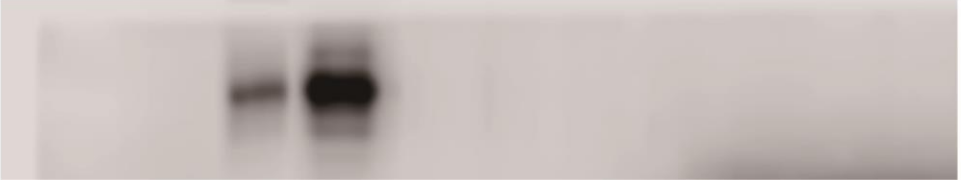

Fig. 7E AAV-GFP-TTR

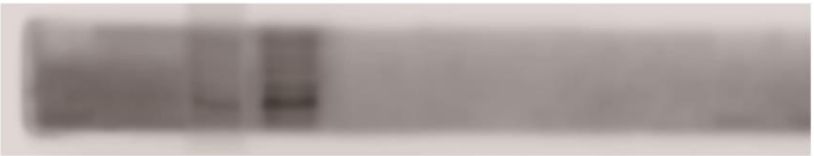

Fig. 7E AAV-GHR-TTR

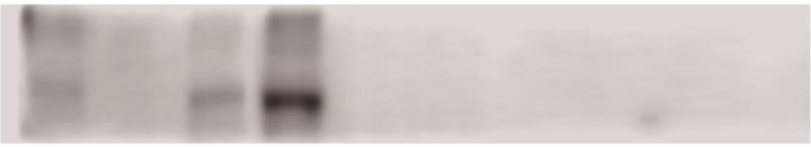

Fig. 7F HIF1α

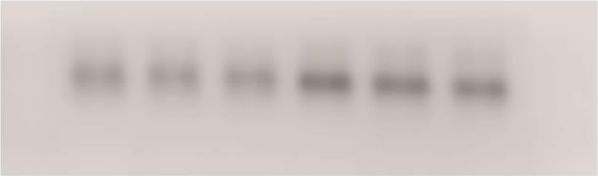

Fig. 7F β-actin

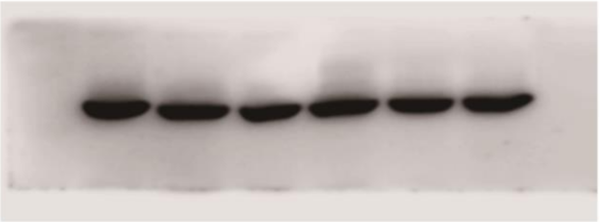

Fig. 7H GHR

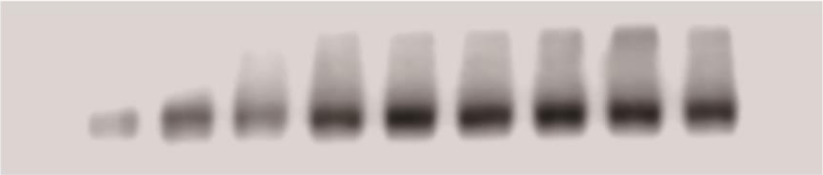

Fig. 7H TTR

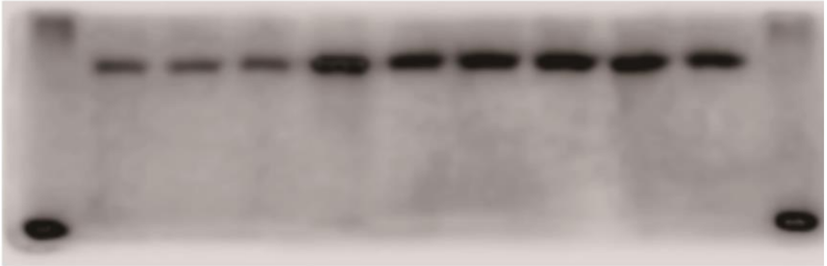

Fig. 7H HIF1α

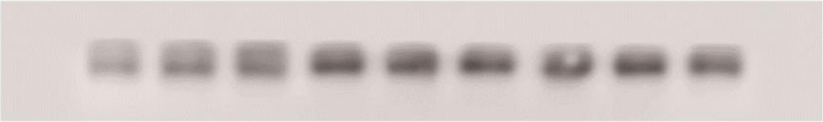

Fig. 7H β-actin

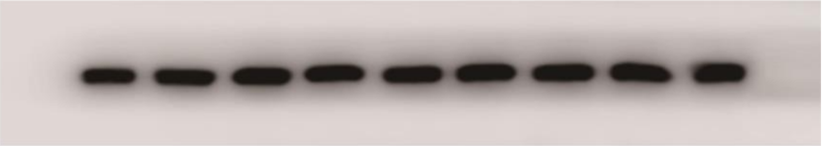

Fig. S7A HIF1α

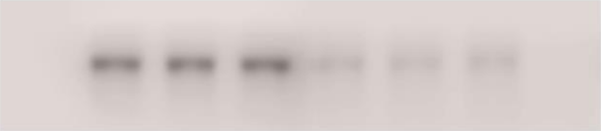

Fig. S7A GAPDH

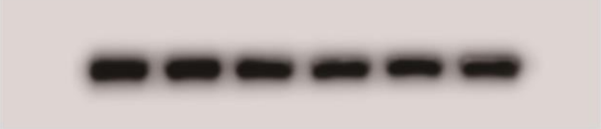

Fig. S7C WT-RBP4

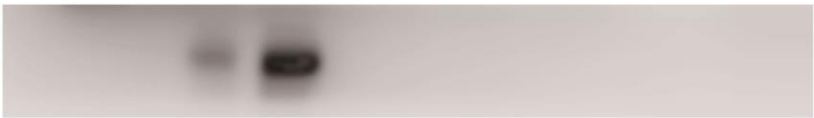

Fig. S7C KO-RBP4

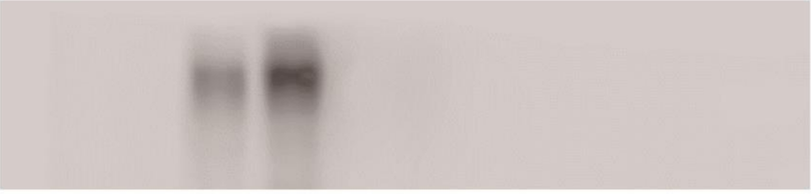

Fig. S7C WT-TTR

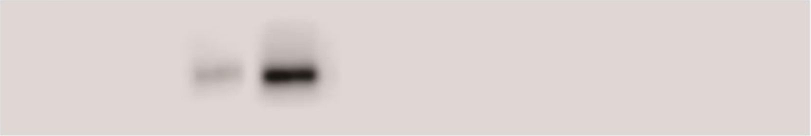

Fig. S7C KO-TTR

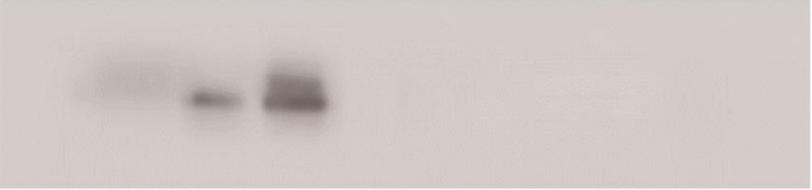

Supplement: Supplementary file 1 — Supplementary figures and tables. [file thnov11p8283s1.pdf]
